# Research Updates and Advances on Flavonoids Derived from Dandelion and Their Antioxidant Activities

**DOI:** 10.3390/antiox13121449

**Published:** 2024-11-25

**Authors:** Xiaocui Zhuang, Wei Shi, Tao Shen, Xiaoyang Cheng, Qilin Wan, Minxia Fan, Dongbao Hu

**Affiliations:** 1 School of Chemical Biology and Environment, Yuxi Normal University, Yuxi 653100, China; zhuangxiaocui@yxnu.edu.cn (X.Z.); weishi@yxnu.edu.cn (W.S.); st@yxnu.edu.cn (T.S.); 2021143102@yxnu.edu.cn (X.C.); 2021143124@yxnu.edu.cn (Q.W.); 2 Key Laboratory of Plant Germplasm Enhancement and Specialty Agriculture, Wuhan Botanical Garden, Chinese Academy of Sciences, Wuhan 430074, China; fanminxia@wbgcas.cn

**Keywords:** dandelion, *Taraxacum* species, flavonoids, antioxidant activities

## Abstract

As a common medicinal and edible plant, dandelion plays a crucial and significant role in the fields of traditional Chinese medicines, functional foods, healthcare products, daily chemicals, and feed additives, which are closely related to its rich chemical constituents and remarkable biological activities. Modern studies have demonstrated that dandelion contains all kinds of bioactive constituents, including flavonoids, amino acids, fatty acids, organic acids, phenolic acids, coumarins, lignans, polysaccharides, phytosterols, terpenes, glycoproteins, oligosaccharides, alkaloids, etc. Meanwhile, dandelion has been proven to possess antioxidant, antibacterial, anti-inflammatory, antitumor, antivirus, hypoglycemic, and hypolipidemic properties, as well as the ability to regulate hormone levels and protect some visceral organs. Among them, flavonoids derived from dandelion and their antioxidant activities have received considerable attention. This study reviews dandelion flavonoids and their in vitro and in vivo antioxidant activities by consulting and organizing relevant domestic and international works of literature to provide a scientific and theoretical basis for further research, development, and utilization of dandelion.

## 1. Introduction

Dandelion is a kind of nutritious wild vegetable, also named *Taraxaci Herba* by the 2020 edition of the *Pharmacopoeia of the People’s Republic of China*, as well as a general term used for various perennial herbaceous plants in the genus *Taraxacum*, family Asteraceae [1,2]. As a traditional Chinese medicine, dandelion is well known for its bitter and sweet taste, cold nature, and its ability to enter the liver and stomach meridians. It has properties of clearing heat and detoxifying, reducing swelling and dissipating nodules, promoting diuresis, and relieving stranguries. It is used to treat symptoms or diseases, such as boils, carbuncles, mastitis, scrofula, red eyes, sore throat, lung abscess, intestinal abscess, damp-heat jaundice, and stranguries with painful urination [1], earning a good reputation as the “queen of medicinal herbs [3]”. To date, modern studies have shown that dandelion contains all kinds of bioactive constituents, including flavonoids, amino acids, fatty acids, organic acids, phenolic acids, coumarins, lignans, polysaccharides, phytosterols, terpenes, glycoproteins, oligosaccharides, alkaloids, etc. Moreover, it has been proven to possess antioxidant, antibacterial, anti-inflammatory, antitumor, antivirus, hypoglycemic, and hypolipidemic properties, as well as the ability to regulate hormone levels and protect some visceral organs, like the liver, gastrointestinal tract, and prostate [4,5,6,7]. As a dual-use plant, dandelion can be processed into beverages, like tea, yogurt, functional drinks, and coffee; foods, like steamed buns, biscuits, bread, pickles, and tofu; and daily chemicals, like toothpaste, facial cleansers, and essential oils, which shows considerable promise for development [2,8,9]. Additionally, dandelion can be used as a green and safe feed additive, replacing antibiotics, incorporated into livestock and poultry feeds to promote animal growth, enhance animal immune function, and improve the quality of animal products [10,11,12]. In conclusion, as a common medicinal and edible plant, dandelion is essential in the fields of traditional Chinese medicines, functional foods, healthcare products, daily chemicals, and feed additives. What’s more, most of these applications are closely related to the antioxidant activities of dandelion [4,9,13,14,15].

Modern studies have indicated that the excessive accumulation of reactive oxygen species (ROS), such as superoxide anion radicals (O_2_^•−^), hydroxyl radicals (·OH), and hydrogen peroxide (H_2_O_2_), in the body can damage multiple tissues and organs, which are closely associated with the onset, development, and prognosis of various diseases, including cardiovascular diseases [16], chronic obstructive pulmonary disease (COPD) [17], schizophrenia [16], Alzheimer’s disease [18], Parkinson’s disease [19,20], non-alcoholic fatty liver disease [21,22], cancer and aging [16], etc. Therefore, scavenging free radicals and enhancing antioxidant activities are crucial for the treatment of these diseases and have significant biological importance for humans [16,23]. In recent years, as a special natural antioxidant, flavonoids, which possess unique advantages of safety and low toxicity, have always been a prominent focus in antioxidant research [24,25]. Dandelion is rich in natural resources, and its flavonoids possess notable antioxidant activities, warranting significant attention [26,27].

Therefore, a comprehensive review of the flavonoids derived from dandelion and their antioxidant activities is necessary, considering its numerous benefits. This study reviews the research progress on flavonoids and their antioxidant activities in dandelion, aiming to provide a reference for further research and the development of dandelion resources.

## 2. Materials and Methods

This review was performed by searching for literature in databases, such as Web of Science, SciFinder, PubMed, Google Scholar, Baidu Scholar, China National Knowledge Infrastructure, WanFang Med Online, and VIP Database, as well as *Pharmacopoeia of the Peoples Republic of China*, with keywords dandelion, *Taraxacum*, constituents, flavonoids, activities, antioxidant, respectively. Subsequently, this review was written by organizing and summarizing the obtained literature.

## 3. Flavonoids

**Figure 1:**
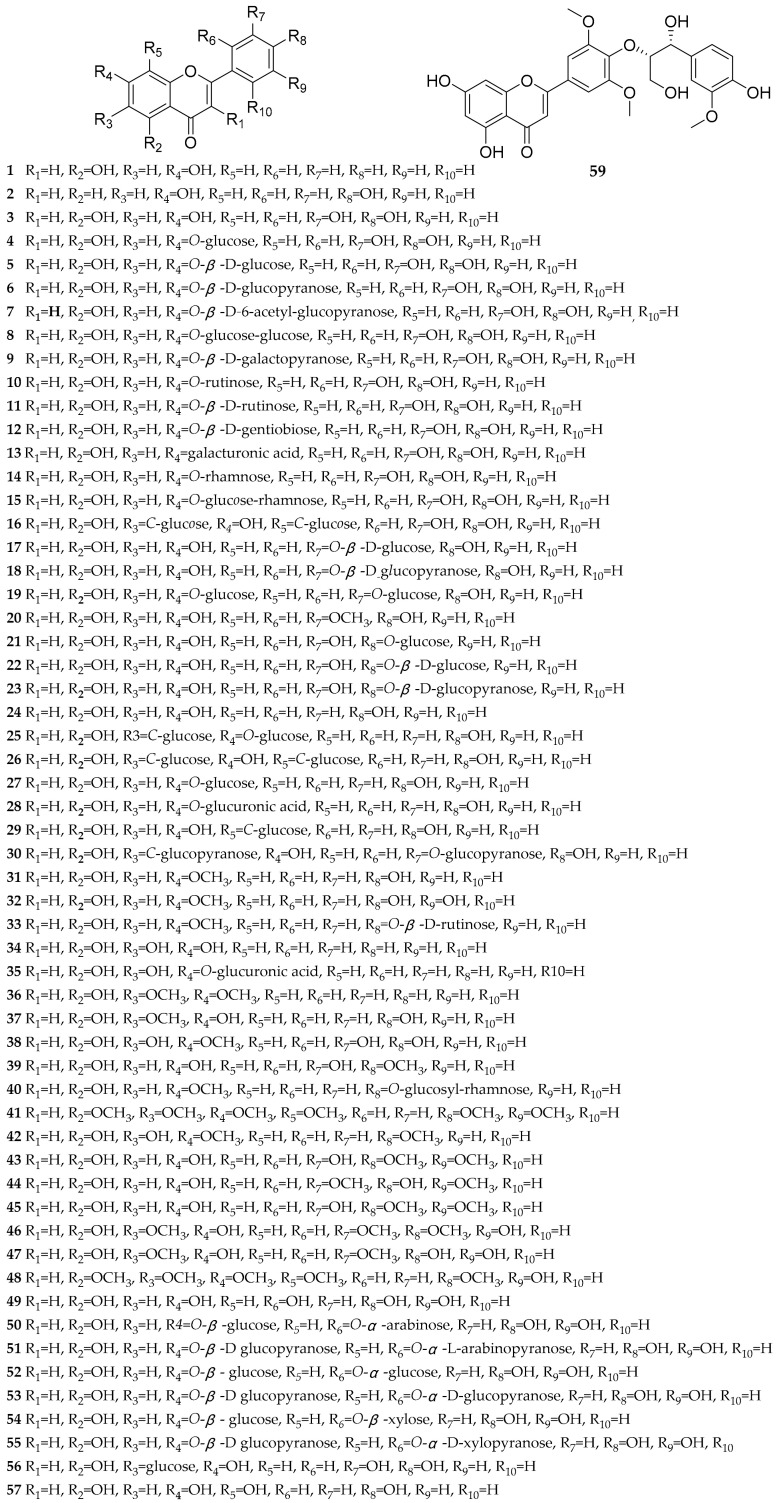
The structures of flavones in dandelion.

**Figure 2:**
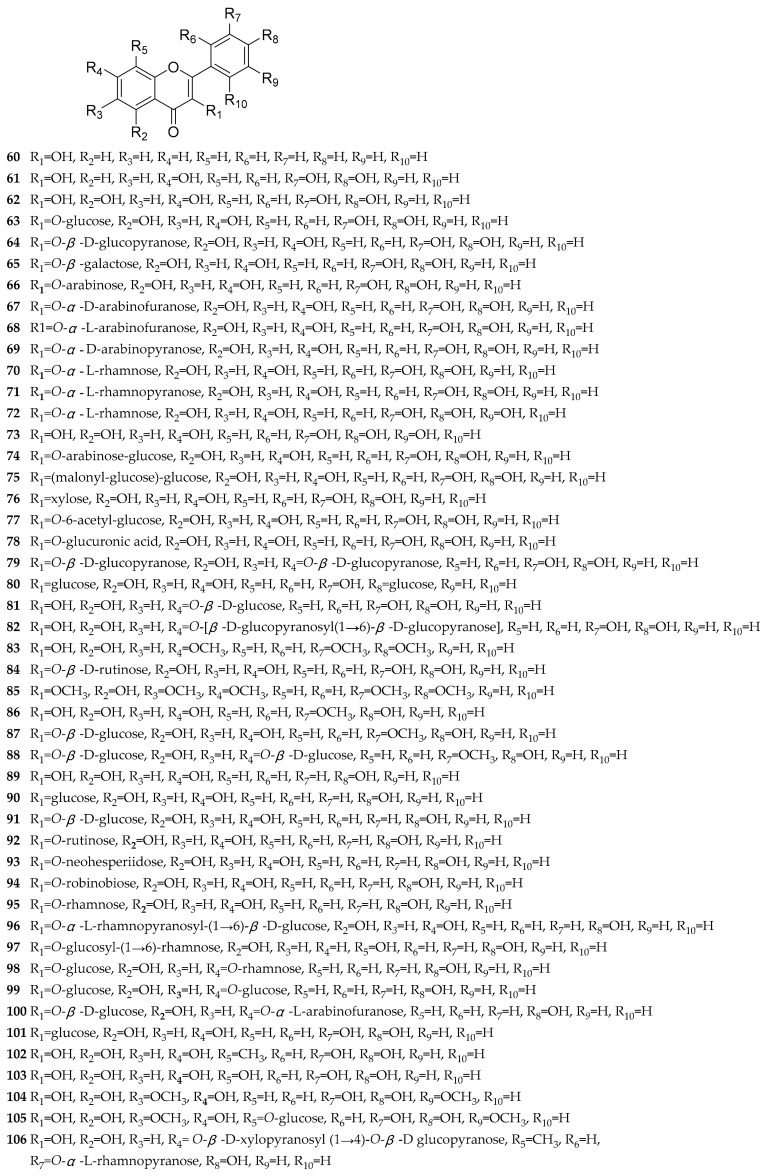
The structures of flavonols in dandelion.

**Figure 3:**
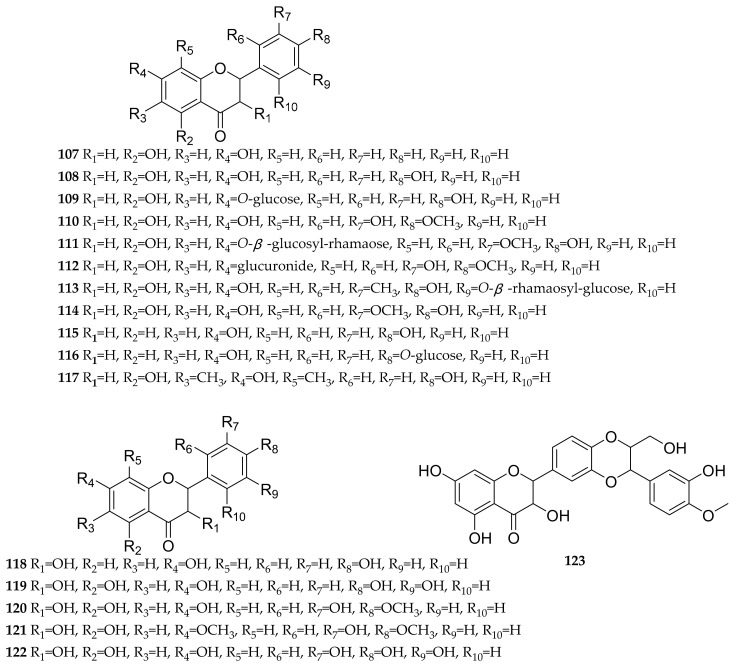
The structures of flavanones and flavanonols in dandelion.

**Figure 4:**
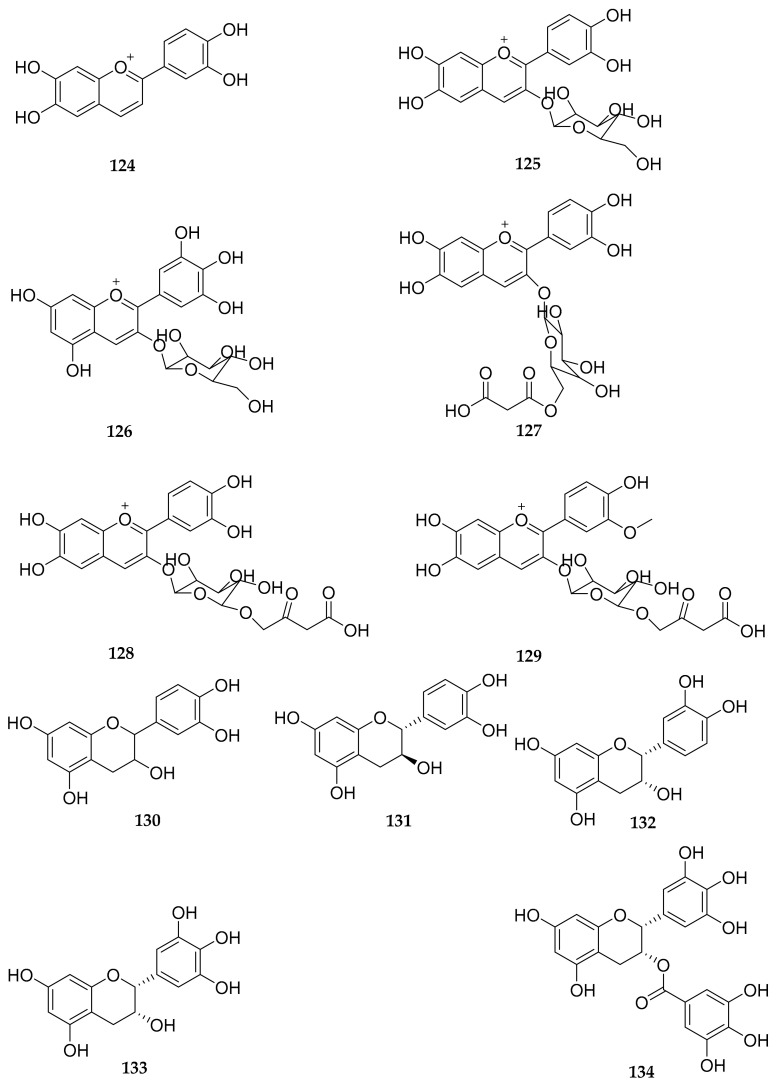
The structures of anthocyanidins and flavan-3-ols in dandelion.

**Figure 5:**
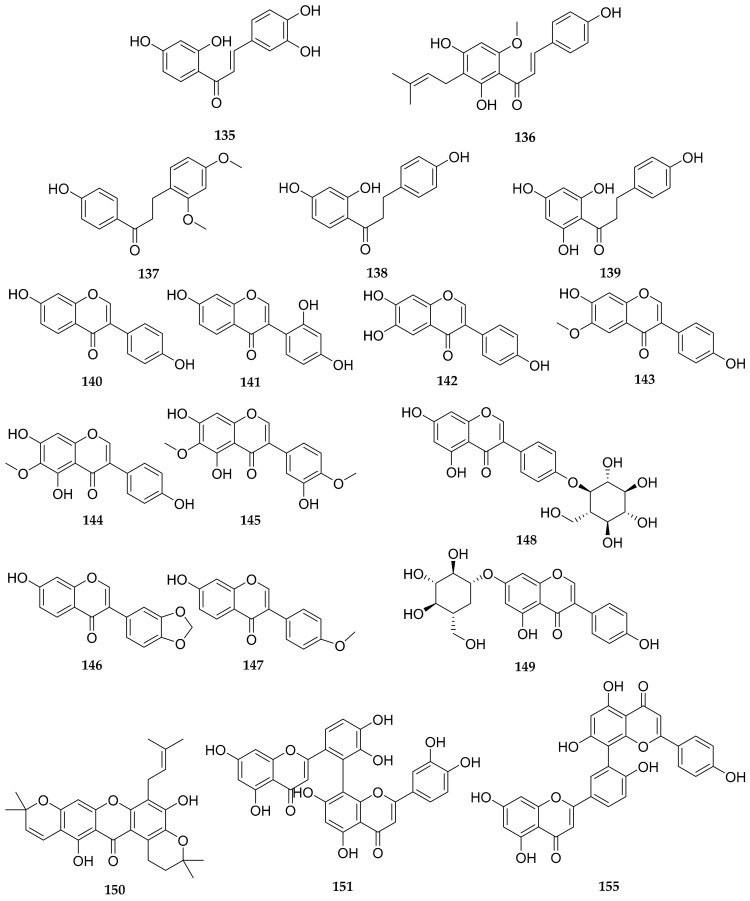
The structures of chalcones, dihydrochalcones, isoflavones, xanthones and biflavonoids in dandelion.

Flavonoids are a class of natural products with a basic C_6_-C_3_-C_6_ skeleton, widely present in the plant kingdom. They are abundant bioactive components in dandelion, exhibiting antioxidant [28,29], antitumor [28], anti-inflammatory activities [30], etc. To date, almost 155 reported flavonoids have been reported in dandelion, mainly including flavones [26,27,29,31,32,33,34,35,36,37,38,39,40,41,42,43,44,45,46,47,48,49,50,51,52,53,54,55,56,57,58,59,60,61,62,63,64,65,66,67,68,69,70,71,72,73,74], flavonols [26,27,29,32,33,34,35,37,39,40,41,43,44,45,52,53,55,56,60,66,68,69,70,73,74,75,76,77,78,79], flavanones [26,29,31,33,43,49,52,68,69,70,80,81], flavanonols [54,60,68,73], anthocyanidins [68,77,82,83], flavan-3-ols [44,84], chalcones [68], dihydrochalcones [55,68,73], isoflavones [55,68,69,70,79], xanthones [78], and bioflavonoids [67,68]. The structures of these compounds are shown in Figure 1, Figure 2, Figure 3, Figure 4 and Figure 5, and their names, sources, and parts are shown in Table 1, Table 2, Table 3, Table 4 and Table 5.

### 3.1. Flavones

According to literature reports, 59 flavones and their glycosides were identified from dandelion (Figure 1 and Table 1), including the following: chrysin (**1**), 7,4′-dihydroxyflavone (**2**), 6,2′-dihydroxyflavone (**3**), luteolin (**4**), luteolin-7-*O*-glucoside (**5**), luteolin-7-*O*-*β*-D-glucoside (**6**), luteolin-7-*O*-*β*-D-glucopyranoside (**7**), luteolin-7-*O*-*β*-D-(6″-acetyl)-glucopyranoside (**8**), luteolin-7-diglucoside (**9**), luteolin-7-*O*-*β*-D-galactopyranoside (**10**), luteolin-7-*O*-rutinose (**11**), luteolin-7-*O*-*β*-D-rutinoside (**12**), luteolin-7-*O*-*β*-D-gentiobioside (**13**), luteolin-7-galacturonide (**14**), luteolin-7-*O*-rhamnoside (**15**), lonicerin (**16**), luteolin-6,8-di-*C*-glucoside (**17**), luteolin-3′-*O*-*β*-D-glucoside (**18**), luteolin-3′-*O*-*β*-D-glucopyranoside (**19**), luteolin-3′,7-*O*-diglucoside (**20**), luteolin-3′-methyl ether (**21**), luteolin-4′-*O*-glucoside (**22**), luteolin-4′-*O*-*β*-D-glucoside (**23**), luteolin-4′-*O*-*β*-D-glucopyranoside (**24**), apigenin (**25**), apigenin-6-*C*-glucoside-7-*O*-glucoside (**26**), apigenin-6,8-di-*C*-glucoside (**27**), apigenin-7-*O*-glucoside (**28**), apigenin-7-*O*-glucuronide (**29**), vitexin (**30**), isovitexin-3″-*O*-glucopyranoside (**31**), genkwanin (**32**), hydroxygenkwanin (**33**), genkwanin-4′-*O*-*β*-D-lutinoside (**34**), baicalein (**35**), 5-hydroxy-6,7-dimethoxyflavonoid (**36**), hispidulin (**37**), pedalitin (**38**), diosmetin (**39**), alquds (**40**), nobiletin (**41**), ladanein (**42**), 5,7,3′-trihydroxy-4′,5′-dimethoxy flavone (**43**), tricin (**44**), apometzgerin (**45**), eupatilin (**46**), jaceosidin (**47**), tangeretin (**48**), isoetin (**49**), isoetin-7-*O*-*β*-D-glucoside-2′-*O*-*α*-arabinoside (**50**), isoetin-7-*O*-*β*-D-glucopyranosyl-2′-*O*-*α*-L-arabinopyranoside (**51**), isoetin-7-*O*-*β*-D-glucoside-2′-*O*-*α*-glucoside (**52**), isoetin-7-*O*-*β*-D-glucopyranosyl-2′-*O*-*α*-D-glucopyranoside (**53**), isoetin-7-*O*-*β*-D-glucoside-2′-*O*-*β*-xyloside (**54**), isoetin-7-*O*-*β*-D-glucopyranosyl-2′-*O*-*β*-D-xylopyranoside (**55**), homoorientin (**56**), isoscutellarein (**57**), tetrahydroxyflavonoe-*C*-rhamnosyl-glucoside (**58**), and salcolin A/B (**59**). 

In summary, the discovery of these compounds was primarily concentrated in whole herbs, roots, flowers, leaves, aerial parts, or vegetative parts of dandelion species, such as *T. mongolicum* Hand.-Mazz, *T. officinale*, *T. officinale* aggregate, *T. officinale* (L.) Weber ex F.H. Wigg, *Neo-T. siphonanthum*, *T. falcilobum*, *T. formosanum* Kitam, *T. sect. Ruderalia*, *T. sinicum* Kitag, *T. extractum*, *T. coreanum*, *T. ohwianum*, or *T. kok-saghyz* Rodin [26,27,29,31,32,33,34,35,36,37,38,39,40,41,42,43,44,45,46,47,48,49,50,51,52,53,54,55,56,57,58,59,60,61,62,63,64,65,66,67,68,69,70,71,72,73,74]. Notably, as indicated in Table 6, chrysin (**1**), 7,4′-dihydroxyflavone (**2**), 6,2′-dihydroxyflavone (**3**), luteolin-7-*O*-glucoside (**5**), luteolin-7-*O*-*β*-D-(6″-acetyl)-glucopyranoside (**8**), luteolin-7-*O*-rutinose (**11**), lonicerin (**16**), luteolin-6,8-di-*C*-glucoside (**17**), apigenin-6-*C*-glucoside-7-*O*-glucoside (**26**), apigenin-6,8-di-*C*-glucoside (**27**), apigenin-7-*O*-glucuronide (**29**), vitexin (**30**), isovitexin-3″-*O*-glucopyranoside (**31**), pedalitin (**38**), tricin (**44**), apometzgerin (**45**), eupatilin (**46**), jaceosidin (**47**), tangeretin (**48**), homoorientin (**56**), isoscutellarein (**57**), tetrahydroxyflavonoe-*C*-rhamnosyl-glucoside (**58**), and salcolin A/B (**59**). They were quickly identified from dandelion by using mass spectrometry [66,67,68,69,70,71,85].

### 3.2. Flavanols

According to literature reports, 47 flavonols and their glycosides were identified from dandelion (Figure 2 and Table 2), including the following: 3-hydroxyflavone (**60**), fisetin (**61**), quercetin (**62**), quercetin-3-*O*-glucoside (**63**), quercetin-3-*O*-*β*-D-glucopyranoside (**64**), quercetin-3-*O*-*β*-galactoside (**65**), quercetin-3-*O*-arabinoside (**66**), quercetin-3-*O*-*α*-D-arabinofuranoside (**67**), avicularin (**68**), quercetin-3-*O*-*α*-D-arabinopyranoside (**69**), quercetin-3-*O*-*α*-L-rhamnoside (**70**), quercetin-3-*O*-*α*-L-rhamnopyranoside (**71**), myricitrin (**72**), myricetin (**73**), quercetin-3-*O*-arabinose-glucoside (**74**), quercetin-3-(malonyl-glucoside)-glucoside (**75**), reynoutrin (**76**), quercetin-3-*O*-(6″-acetyl-glucoside) (**77**), quercetin-3-*O*-glucuronide (**78**), quercetin-3,7-*O*-*β*-D-diglucopyranoside (**79**), quercetin-3,4′-diglucoside (**80**), quercetin-7-*O*-*β*-D-glucoside (**81**), quercetin-7-*O*-[*β*-D-glucopyranosyl (1→6)-*β*-D-glucopyranoside] (**82**), quercetin-3′,4′,7-trimethyl ether (**83**), rutin (**84**), artemetin (**85**), isorhamnetin (**86**), isorhamnetin-3-*O*-*β*-D-glucoside (**87**), isorhamnetin-3-*O*-*β*-D-glucoside (**88**), kaempferol (**89**), kaempferol-3-glucoside (**90**), kaempferol-3-*O*-*β*-D-glucopyranoside (**91**), kaempferol-3-*O*-rutinoside (**92**), kaempferol-3-*O*-neohesperiidoside (**93**), kaempferol-3-*O*-robinobioside (**94**), kaempferol-3-*O*-rhamnoside (**95**), kaempferol-3-*O*-*α*-L-rhamnopyranoside-(1→6)-*β*-D-glucoside (**96**), nicotiflorin (**97**), kaempferol-3-*O*-glucoside-7-*O*-rhamnoside (**98**), kaemperfol-3,7-diglucoside (**99**), kaempferol-3-*O*-*β*-D-glucoside-7-*O*-*α*-L-arabinofuranoside (**100**), hyperseroside (**101**), 3,5,7,3′,4′-pentahydroxy-8-*C*-methylflavone (**102**), 2-(3,4-dihydroxy-5-methoxyphenyl)-3,5,7-trihydroxy-6-methoxy-4*H*-chromen-4-one (**103**), gossypetin (**104**), gossypetin-8-*O*-glucoside (**105**), and 3,5,7,3′,4′-pentahydroxy-8-*C*-methylflavone-7-*O*-*β*-D-xylopyranosyl-(1→4)-*O*-*β*-D-glucopyranosyl-3′-*O*-*α*-L-rhamnopyranoside (**106**).

Moreover, the discovery of these compounds was primarily concentrated in whole herbs, aerial parts, flowers, leaves, roots, or stems of dandelion species, such as *T. mongolicum* Hand.-Mazz, *T. officinale*, *T. officinale* (L.) Weber ex F.H. Wigg, *Neo-T. siphonanthum*, *T. brevicorniculatum*, *T. bessarabicum*, *T. sinicum* Kitag, *T. extractum*, *T. coreanum*, or *T. kok-saghyz* Rodin [26,27,29,32,33,34,35,37,39,40,41,43,44,45,52,53,55,56,60,66,68,69,70,73,74,75,76,77,78,79]. Obviously, as shown in Table 6, 3-hydroxyflavone (**60**), quercetin-3-*O*-glucoside (**63**), avicularin (**68**), quercetin-3-*O*-*α*-L-rhamnopyranoside (**71**), myricitrin (**72**), myricetin (**73**), reynoutrin (**76**), quercetin-3-*O*-(6″-acetyl-glucoside) (**77**), quercetin-3-*O*-glucuronide (**78**), rutin (**84**), kaempferol-3-*O*-*β*-D-glucopyranoside (**91**), kaempferol-3-*O*-rutinoside (**92**), kaempferol-3-*O*-neohesperiidoside (**93**), kaempferol-3-*O*-robinobioside (**94**), kaempferol-3-*O*-rhamnoside (**95**), kaempferol-3-*O*-glucoside-7-*O*-rhamnoside (**98**), kaemperfol-3,7-diglucoside (**99**), kaempferol-3-*O*-*β*-D-glucoside-7-*O*-*α*-L-arabinofuranoside (**100**), and gossypetin-8-*O*-glucoside (**105**). These were quickly identified from dandelion by using mass spectrometry [66,68,69,70,73].

### 3.3. Flavanones

According to the current literature reports, 11 flavanones and their glycosides have been identified from dandelion, including the following: 5,7-dihydroxyflavanone (**107**), naringenin (**108**), naringenin-7-*O*-glucoside (**109**), hesperetin (**110**), hesperidin (**111**), hesperetin-7-glucuronide (**112**), hesperetin-5′-*O*-*β*-rhamnoglucoside (**113**), 4′,5,7-trihydroxy-3′-methoxy-dihydroflavone (**114**), liquiritigenin (**115**), liquiritin (**116**), and farrerol (**117**) (Figure 3 and Table 3). 

Obviously, the discovery of these compounds was primarily concentrated in whole herbs, leaves, or aerial parts of dandelion species, such as *T. mongolicum* Hand.-Mazz, *T. officinale*, or *T. extractum* [26,29,31,33,43,49,52,68,69,70,80,81]. 5,7-Dihydroxyflavanone (**107**), naringenin (**108**), hesperetin (**110**), hesperidin (**111**), hesperetin-7-glucuronide (**112**), and hesperetin-5′-*O*-*β*-rhamnoglucoside (**113**) were derived from the whole herbs of *T. mongolicum* Hand.-Mazz [26,29,31,33,43,49,52,54,69]. Notably, as displayed in Table 6, naringenin (**108**), naringenin-7-*O*-glucoside (**109**), liquiritigenin (**115**), liquiritin (**116**), and farrerol (**117**), as quickly identified from dandelion by using mass spectrometry [68,69,70].

### 3.4. Flavanonols

According to the current literature reports, six flavanonols and their glycosides have been identified from dandelion, including the following: garbanzol (**118**), toxifolin (**119**), (2*R*,3*R*)-(+)-4′-*O*-methyldihydro-quercetin (**120**), (2*R*,3*R*)-(+)-4′,7-di-*O*-methyldihydro-quercetin (**121**), dihydromyricetin (**122**) and silymarin (**123**) (Figure 3 and Table 3). 

Obviously, the discovery of these compounds was primarily concentrated in whole herbs or roots of dandelion species, such as *T. mongolicum* Hand.-Mazz, *T. officinale*, *Neo-T. siphonanthum*, or *Taraxaci Herba* [54,60,68,73]. Notably, as presented in Table 6, garbanzol (**118**), toxifolin (**119**), dihydromyricetin (**122**) and silymarin (**123**) were also quickly identified from dandelion via mass spectrometry [54,68,73].

### 3.5. Anthocyanidins

According to the current literature reports, six anthocyanidins and their glycosides have been identified from dandelion, including the following: cyanidin (**124**), cyanidin-3-glucoside (**125**), delphinidin-3-*O*-glucoside (**126**), cyanidin-3-(6-malonyl)-glucoside (A-1) (**127**), cyanidin-3-(6-malonyl)-glucoside (A-2) (**128**), peonidin-3-(6-malonyl)-glucoside (**129**) (Figure 4 and Table 4). As demonstrated in Table 6, cyanidin (**124**) and delphinidin-3-*O*-glucoside (**126**) were identified from whole herbs of unfermented and fermented *T. officinale*, respectively [68]. Four anthocyanins, including cyanidin-3-glucoside (**125**), cyanidin-3-(6-malonyl)-glucoside (A-1) (**127**), cyanidin-3-(6-malonyl)-glucoside (A-2) (**128**) and peonidin-3-(malonyl)-glucoside (**129**), were identified from leaves of *T. officinale* through liquid chromatography electrospray ionization with high-resolution quadrupole time-of-flight mass spectrometry (LC-ESI-HR-QTOF-MS) for the first time [83]. 

### 3.6. Flavan-3-ols

The main flavan-3-ols identified from dandelion were catechin (**130**), (+)-catechin (**131**), (−)-epicatechin (**132**), (−)-epigallocatechin (**133**), and (−)-epigallocatechingallate (**134**) (Figure 4 and Table 4). Actually, as exhibited in Table 6, there were 10 active substances determined by using high-performance liquid chromatography (HPLC) from dandelion. The mass concentration of catechin (**130**) in the range of 10.0–100 µg/mL showed a good linear relationship with the peak area, with correlation coefficients not less than 0.995 and detection limits of 1.87–15.9 μg/g [44]. (+)-Catechin (**131**), (−)-epicatechin (**132**), (−)-epigallocatechin (**133**), and (−)-epigallocatechingallate (**134**) were analyzed from *T. officinale* and other herb teas [84].

### 3.7. Chalcones

There were two chalcones identified from dandelion. They were butein (**135**) and xanthohumol (**136**). As exhibited in Table 6, butein (**135**) and xanthohumol (**136**) were identified from whole herbs of fermented *T. officinale* by liquid chromatography electrospray ionization mass spectrometry (LC-ESI-MS/MS) [68].

### 3.8. Dihydrochalcones

There were three dihydrochalcones identified from dandelion. They were loureirin A (**137**), isoliquiritigenin (**138**) and phloretin (**139**). As illustrated in Table 6, loureirin A (**137**) was identified from *T. kok-saghyz* Rodin by ultra-high performance liquid chromatography-tandem hybrid quadrupole-electrostatic field orbitrap high resolution mass spectrometry (UHPLC-Q/Orbitrap HRMS) [55]. Isoliquiritigenin (**138**) was identified from whole herbs of fermented *T. officinale* by LC-ESI-MS/MS [68]. Phloretin (**139**) was isolated and characterized from fermented *T. mongolicum* Hand.-Mazz [73].

### 3.9. Isoflavones

There were 10 isoflavones identified from dandelion. They were daidzein (**140**), 2-hydroxyxydaidzein (**141**), genistein (**142**), glycitein (**143**), tectorigenin (**144**), iristectorigenina (**145**), pseudobaptigenin (**146**), formononetin (**147**), sophoricoside (**148**), and genistin (**149**). As indicated in Table 6, tectorigenin (**144**) and iristectorigenina (**145**), identified from *T. kok-saghyz* Rodin by UHPLC-Q/Orbitrap HRMS [55]. Daidzein (**140**) and 2-hydroxyxydaidzein (**141**) were identified from whole herbs of fermented *T. officinale* by LC-ESI-MS/MS [68]. Genistein (**142**) and glycitein (**143**) were identified from *T. mongolicum* Hand.-Mazz., produced in Gansu and Jiangsu province in China, by high performance liquid chromatography-tandem hybrid quadrupole-electrostatic field orbitrap high resolution mass spectrometry (HPLC-QTOF-MS) [69]. Genistein (**142**), tectorigenin (**144**), pseudobaptigenin (**146**), formononetin (**147**), and genistin (**149**) were identified from *T. extractum* by ultra-high performance liquid chromatography with high-resolution mass spectrometry (UHPLC-HRMS/MS) [70]. Sophoricoside (**148**) was identified from aerial parts of *T. coreanum* Nakai by ultra-liquid chromatography electrospray ionization mass spectrometry (UHPLC-ESI-MS) [79].

### 3.10. Xanthones

As shown in Table 6, the main xanthone mangostenone B (**150**) was quickly identified from dandelion extract purchased from Zeland Co., Ltd. (Nanjing, China) via ultra-high performance liquid chromatography coupled with quadrupole time of flight mass spectrometry (UPLC-QTOF MS) [78].

### 3.11. Biflavonoids

The main biflavonoids identified from dandelion were philonotisflavone (**151**), luteolin-luteolin (**152**), luteolin-apigenin (**153**), luteolin-chrysoeriol (**154**) and amentoflavone (**155**) (Figure 5 and Table 5). As manifested in Table 6, philonotisflavone (**151**), luteolin-luteolin (**152**), luteolin-apigenin (**153**) and luteolin-chrysoeriol (**154**) were quickly identified from *T. officinale* L. fruits by ultra-high performance liquid chromatography coupled with diode array detector, corona-charged aerosol detector, and quadrupole time of flight mass spectrometry (UHPLC-PDA-CAD-ESI-QTOF-MS/MS) [67]. Amentoflavone (**155**) was identified from whole herbs of *T. officinale* by LC-ESI-MS/MS [68].

**Table 1:** Flavones identified from dandelion.

No.	Compounds	*Taraxacum* Species	Parts	References
1	chrysin	*Taraxacum mongolicum* Hand.-Mazz	whole herbs	[69]
2	7,4′-dihydroxyflavone	fermented *Taraxacum officinale*	whole herbs	[68]
3	6,2′-dihydroxyflavone	*Taraxacum extractum*	aerial parts	[70]
4	luteolin	*Taraxacum mongolicum* Hand.-Mazz	whole herbs	[26,27,33,34,43]
		*Taraxacum mongolicum* Hand.-Mazz	aerial parts	[29,40]
		*Taraxacum mongolicum* Hand.-Mazz	flowers	[45]
		*Taraxacum officinale* aggregate	flowers and leaves	[36]
		*Taraxacum sinicum* Kitag.	whole herbs	[38]
		*Neo-Taraxacum siphonathum*	whole herbs	[39]
		*Taraxacum* sect. *Ruderalia*	flowers and vegetative parts	[47]
		*Taraxacum coreanum*	roots, leaves and flowers	[62]
		*Taraxacum ohwianum*	roots, leaves and flowers	[62]
		*Taraxacum officinale*	roots, leaves and flowers	[62]
5	luteolin-7-*O*-glucoside	*Taraxacum mongolicum* Hand.-Mazz	whole herbs	[26,27,35,41]
		*Taraxacum sinicum* Kitag.	whole herbs	[32]
		*Taraxacum formosanum* Kitam	whole herbs	[46]
		*Taraxacum officinale* WEB. ex WIGG.	roots and herbs juice	[48]
		*Taraxacum* sect. *Ruderalia*	flowers and vegetative parts	[47]
		*Taraxacum falcilobum*	whole herbs	[42]
		*Taraxacum extractum*	aerial parts	[70]
6	luteolin-7-*O*-*β*-D-glucoside	*Taraxacum mongolicum* Hand.-Mazz	whole herbs	[26,33,34,43]
		*Taraxacum mongolicum* Hand.-Mazz	flowers	[45]
		*Taraxacum coreanum*	roots, leaves and flowers	[62]
		*Taraxacum ohwianum*	roots, leaves and flowers	[62]
		*Taraxacum officinale*	roots, leaves and flowers	[62]
7	luteolin-7-*O*-*β*-D-glucopyranoside	*Taraxacum mongolicum* Hand.-Mazz	aerial parts	[29]
		*Neo-Taraxacum siphonathum*	whole herbs	[39,59]
8	luteolin-7-*O*-*β*-D-(6″-acetyl)-glucopyranoside	*Taraxacum officinale*	leaves	[66]
9	luteolin-7-diglucoside	*Taraxacum officinale* aggregate	flowers and leaves	[36]
10	luteolin-7-*O*-*β*-D-galactopyranoside	*Taraxacum mongolicum* Hand.-Mazz	whole herbs	[26,29,31,33,43]
		*Taraxacum mongolicum* Hand.-Mazz	aerial parts	[29]
11	luteolin-7-*O*-rutinoside	*Taraxacum formosanum* Kitam	whole herbs	[46]
		*Taraxacum officinale* WEB. ex WIGG.	roots and herbs juice	[48]
		*Taraxacum officinale*	leaves	[65]
		*Taraxacum* sect. *Ruderalia*	flowers and vegetative parts	[47]
		*Taraxacum extractum*	aerial parts	[70]
12	luteolin-7-*O*-*β*-D-rutinoside	*Taraxacum mongolicum* Hand.-Mazz	flowers	[26,45]
13	luteolin-7-*O*-*β*-D-gentiobioside	*Taraxacum mongolicum* Hand.-Mazz	flowers	[26,45]
14	luteolin-7-galacturonide	*Taraxacum mongolicum* Hand.-Mazz	whole herbs	[86]
15	luteolin-7-*O*-rhamnoside	*Taraxacum officinale*	leaves	[74]
16	lonicerin	*Taraxacum mongolicum* Hand.-Mazz	whole herbs	[73]
17	luteolin-6,8-di-*C*-glucoside	*Taraxacum mongolicum* Hand.-Mazz	whole herbs	[71]
18	luteolin-3′-*O*-*β*-D-glucoside	*Taraxacum mongolicum* Hand.-Mazz	flowers	[45]
19	luteolin-3′-*O*-*β*-D-glucopyranoside	*Neo-Taraxacum siphonanthum*	whole herbs	[60,61]
20	luteolin-3′,7-*O*-diglucoside	*Taraxacum officinale*	leaves	[74]
21	chrysoeriol	*Taraxacum officinale* aggregate	flowers and leaves	[26,36]
		*Taraxacum officinale* WEB. ex WIGG.	roots and herbs juice	[48]
22	luteolin-4′-*O*-glucoside	*Taraxacum officinale* WEB. ex WIGG.	roots and herbs juice	[48]
23	luteolin-4′-*O*-*β*-D-glucoside	*Taraxacum mongolicum* Hand.-Mazz	flowers	[45]
24	luteolin-4′-*O*-*β*-D-glucopyranoside	*Neo-Taraxacum siphonanthun*	whole herbs	[59,60]
25	apigenin	*Taraxacum sinicum* Kitag.	whole herbs	[26,27,32]
26	apigenin-6-*C*-glucoside-7-*O*-glucoside	*Taraxacum mongolicum* Hand.-Mazz	whole herbs	[69]
27	apigenin-6,8-di-*C*-glucoside	*Taraxacum mongolicum* Hand.-Mazz	whole herbs	[69]
28	apigenin-7-*O*-glucoside	*Taraxacum sinicum* Kitag.	whole herbs	[26,27,31,32]
29	apigenin-7-*O*-glucuronide	*Taraxacum extractum*	aerial parts	[70]
30	vitexin	*Taraxacum mongolicum* Hand.-Mazz	whole herbs	[69]
		*Taraxacum extractum*	aerial parts	[70]
31	isovitexin-3″-*O*-glucopyranoside	*Taraxacum officinale*	leaves	[66]
32	genkwanin	*Taraxacum mongolicum* Hand.-Maz*z*	whole herbs	[26,29,31,33,43]
		*Taraxacum mongolicum* Hand.-Mazz	aerial parts	[29]
33	hydroxygenkwanin	*Taraxacum kok-saghyz* Rodin	roots and leaves	[55]
34	genkwanin-4′-*O*-*β*-D-lutinoside	*Taraxacum mongolicum* Hand.-Mazz	whole herbs	[26,29,31,33,43]
		*Taraxacum mongolicum* Hand.-Mazz	aerial parts	[29]
		*Neo-Taraxacum siphonanthum*	whole herbs	[60]
35	baicalein	*Taraxacum mongolicum* Hand.-Mazz	whole herbs	[52]
36	5-hydroxy-6,7-dimethoxyflavonoid	*Taraxacum kok-saghyz* Rodin	aerial parts	[55]
37	hispidulin	*Taraxacum kok-saghyz* Rodin	aerial parts	[55]
		*Taraxacum mongolicum* Hand.-Mazz	whole herbs	[73]
38	pedalitin	Fermented *Taraxacum officinale*	whole herbs	[68]
39	diosmetin	*Taraxacum sinicum* Kitag.	whole herbs	[26,38]
40	alquds	*Taraxacum mongolicum* Hand.-Mazz	aerial parts	[49]
41	nobiletin	*Taraxaci Herba*	roots	[54]
42	ladanein	*Taraxacum officinale* (L.) Weber	stems	[53]
43	5,7,3′-trihydroxy-4′,5′-dimethoxy flavone	*Taraxacum officinale* F. H. Wigg	not mentioned	[64]
44	tricin	*Taraxacum officinale* F. H. Wigg	not mentioned	[64]
		*Taraxacum officinale* L.	fruits	[67]
		*Taraxacum mongolicum* Hand.-Mazz	whole herbs	[73]
		*Taraxacum extractum*	aerial parts	[70]
45	apometzgerin	*Taraxacum officinale* L.	fruits	[67]
46	eupatilin	*Taraxacum mongolicum*	whole herbs	[71,72]
47	jaceosidin	*Taraxacum mongolicum* Hand.-Mazz	whole herbs	[73]
48	tangeretin	*Taraxacum mongolicum* Hand.-Mazz	whole herbs	[73]
49	isoetin	*Taraxacum mongolicum* Hand.-Mazz	whole herbs	[29,31,33,43,63]
			aerial parts	[29]
50	isoetin-7-*O*-*β*-D-glucoside-2′-*O*-*α*-arabinoside	*Taraxacum mongolicum* Hand.-Mazz	whole herbs and aerial parts	[63]
51	isoetin-7-*O*-*β*-D-glucopyranosyl-2′-*O*-*α*-L-arabinopyranoside	*Taraxacum mongolicum* Hand.-Mazz	whole herbs	[31,33]
		*Taraxacum mongolicum* Hand.-Mazz	aerial parts	[29,50,51]
52	isoetin-7-*O*-*β*-D-glucoside-2′-*O*-*α*-glucoside	*Taraxacum mongolicum* Hand.-Mazz	whole herbs and aerial parts	[63]
53	isoetin-7-*O*-*β*-D-glucopyranosyl-2′-*O*-*α*-D-glucopyranoside	*Taraxacum mongolicum* Hand.-Mazz	whole herbs	[31,33,43]
		*Taraxacum mongolicum* Hand.-Mazz	aerial parts	[29,50,51]
54	isoetin-7-*O*-*β*-D-glucoside-2′-*O*-*β*-xyloside	*Taraxacum mongolicum* Hand.-Mazz	whole herbs	[63]
		*Taraxacum formosanum*	aerial parts	[63]
55	isoetin-7-*O*-*β*-D-glucopyranosyl-2′-*O*-*β*-D-xyloypyranoside	*Taraxacum mongolicum* Hand.-Mazz	whole herbs	[31,33,43]
		*Taraxacum mongolicum* Hand.-Mazz	aerial parts	[29,50]
56	homoorientin	*Taraxacum mongolicum* Hand.-Mazz	whole herbs	[69,73,86]
57	isoscutellarein	*Taraxacum mongolicum* Hand.-Mazz	whole herbs	[73]
58	tetrahydroxyflavonoe-*C*-rhamnosyl-glucoside	*Taraxacum mongolicum* Hand.-Mazz	whole herbs	[73]
59	salcolin A/B	*Taraxacum officinale* L.	fruits	[67]

Note: *Taraxaci Herba*: The common name of dandelion in the *Pharmacopoeia of the People’s Republic of China*, and the accurate Latin name was not mentioned in the literature.

**Table 2:** Flavonols identified from dandelion.

No.	Compounds	*Taraxacum* Species	Parts	References
60	3-hydroxyflavone	*Taraxacum officinale*	whole herbs	[68]
61	fisetin	*Taraxacum kok-saghyz* Rodin	roots and leaves	[55]
62	quercetin	*Taraxacum mongolicum* Hand.-Mazz	whole herbs	[26,27,29,33,34,35,41]
		*Taraxacum mongolicum* Hand.-Mazz	aerial parts	[43,52]
		*Neo-Taraxacum siphonathum*	whole herbs	[39]
		*Taraxacum mongolicum* Hand.-Mazz	flowers	[45]
63	quercetin-3-*O*-glucoside	*Taraxacum mongolicum* Hand.-Mazz	whole herbs	[26,27,37]
		*Taraxacum extractum*	aerial parts	[70]
64	quercetin-3-*O*-*β*-D-glucopyranoside	*Neo-Taraxacum siphonathum*	whole herbs	[39]
		*Taraxacum coreanum* Nakai	aerial parts	[79]
65	quercetin-3-*O*-*β*-galactoside	*Taraxacum mongolicum* Hand.-Mazz	whole herbs	[26,27,37]
66	quercetin-3-*O*-arabinoside	*Taraxacum officinale*	leaves	[74]
67	quercetin-3-*O*-*α*-D-arabinofuranoside	*Neo-Taraxacum siphonathum*	whole herbs	[26,31,39]
		*Taraxacum coreanum* Nakai	aerial parts	[79]
68	avicularin	*Taraxacum mongolicum* Hand.-Mazz	whole herbs	[73]
69	quercetin-3-*O*-*α*-D-arabinopyranoside	*Neo-Taraxacum siphonathum*	whole herbs	[26,31,39]
		*Taraxacum coreanum* Nakai	aerial parts	[79]
70	quercetin-3-*O*-*α*-L-rhamnoside	*Neo-Taraxacum siphonanthum*	whole herbs	[60]
		*Taraxacum coreanum* Nakai	aerial parts	[79]
71	quercetin-3-*O*-*α*-L-rhamnopyranoside	*Taraxacum mongolicum* Hand.-Mazz	whole herbs	[73]
72	myricitrin	*Taraxacum officinale*	whole herbs	[68]
73	myricetin	*Taraxacum officinale*	leaves	[66]
74	quercetin-3-*O*-arabinose-glucoside	*Taraxacum officinale*	leaves	[74]
		*Taraxacum brevicorniculatum*	leaves	[77]
75	quercetin-3-(malonyl-glucoside)-glucoside	*Taraxacum officinale*	leaves	[74]
76	reynoutrin	*Taraxacum mongolicum* Hand.-Mazz	whole herbs	[69]
77	quercetin-3-*O*-(6″-acetyl-glucoside)	*Taraxacum mongolicum* Hand.-Mazz	whole herbs	[69]
78	quercetin-3-*O*-glucuronide	*Taraxacum extractum*	aerial parts	[70]
79	quercetin-3,7-*O*-*β*-D-diglucopyranoside	*Taraxacum mongolicum* Hand.-Mazz	whole herbs	[26,29,31,33,43]
		*Taraxacum mongolicum* Hand.-Mazz	aerial parts	[29]
80	quercetin-3,4′-diglucoside	*Taraxacum officinale*	leaves	[74]
81	quercetin-7-*O*-*β*-D-glucoside	*Taraxacum mongolicum* Hand.-Mazz	flowers	[26,45]
82	quercetin-7-*O*-[*β*-D-glucopyranosyl-(1→6)-*β*-D-glucopyranoside]	*Taraxacum mongolicum* Hand.-Mazz	whole herbs	[26,29,31,33,43]
		*Taraxacum mongolicum* Hand.-Mazz	aerial parts	[29]
83	quercetin-3′,4′,7-trimethylether	*Taraxacum mongolicum* Hand.-Mazz	whole herbs	[26,29,31,33,43]
		*Taraxacum mongolicum* Hand.-Mazz	aerial parts	[40]
84	rutin	*Taraxacum sinicum* Kitag.	whole herbs	[26,27,31,32]
		*Taraxacum mongolicum* Hand.-Mazz	whole herbs	[52]
		*Taraxacum extractum*	aerial parts	[70]
85	artemetin	*Taraxacum mongolicum* Hand.-Mazz	whole herbs	[26,27,29,31,33,34]
		*Taraxacum mongolicum* Hand.-Mazz	Aerial parts	[29]
86	isorhamnetin	*Taraxacum mongolicum* Hand.-Mazz	whole herbs	[56]
87	isorhamnetin-3-*O*-*β*-D-glucoside	*Taraxacum mongolicum* Hand.-Mazz	flowers	[26,45]
88	isorhamnetin-3,7-*O*-*β*-D-diglucoside	*Taraxacum mongolicum* Hand.-Mazz	flowers	[26,45]
89	kaempferol	*Taraxaci Herba*	not mentioned	[44]
90	kaempferol-3-glucoside	*Taraxacum mongolicum* Hand.-Mazz	whole herbs	[52]
91	kaempferol-3-*O*-*β*-D-glucopyranoside	*Taraxacum officinale*	leaves	[66]
92	kaempferol-3-*O*-rutinoside	*Taraxacum mongolicum* Hand.-Mazz	whole herbs	[69,73]
		*Taraxacum extractum*	aerial parts	[70]
93	kaempferol-3-*O*-neohesperiidoside	*Taraxacum mongolicum* Hand.-Mazz	whole herbs	[73]
94	kaempferol-3-*O*-robinobioside	*Taraxacum mongolicum* Hand.-Mazz	whole herbs	[73]
95	kaempferol-3-*O*-rhamnoside	*Taraxacum officinale*	leaves	[66]
96	kaempferol-3-*O*-*α*-L-rhamnopyranoside-(1→6)-*β*-D-glucoside	*Taraxacum mongolicum* Hand.-Mazz	whole herbs	[75]
97	nicotiflorin	*Taraxacum coreanum* Nakai	aerial parts	[79]
98	kaempferol-3-*O*-glucoside-7-*O*-rhamnoside	*Taraxacum mongolicum* Hand.-Mazz	whole herbs	[73]
99	kaemperfol-3,7-diglucoside	*Taraxacum officinale*	leaves	[66]
100	kaempferol-3-*O*-*β*-D-glucoside-7-*O*-*α*-L-arabinofuranoside	*Taraxacum officinale*	leaves	[66]
101	hyperseroside	*Taraxacum mongolicum* Hand.-Mazz	whole herbs	[52]
102	3,5,7,3′,4′-pentahydroxy 8-*C*-methyl flavone	*Taraxacum officinale* (L.) Weber	stems	[53]
103	2-(3,4-dihydroxy-5-methoxyphenyl)-3,5,7-trihydroxy-6-methoxy-4*H*-chromen-4-one	*Taraxaci Herba*	not mentioned	[78]
104	gossypetin	*Taraxacum bessarabicum*	aerial parts	[76]
105	gossypetin-8-*O*-glucoside	*Taraxacum mongolicum* Hand.-Mazz	whole herbs	[69]
106	3,5,7,3′,4′-pentahydroxy-8-*C*-methyl flavone 7-*O*-*β*-D-xylopyranosyl (1→4)-*O*-*β*-D glucopyranosyl 3′-*O*-*α*-L-rhamnopyranoside	*Taraxacum officinale* (L.) Weber	stems	[31,53]

Note: *Taraxaci Herba*: The common name of dandelion in the *Pharmacopoeia of the People’s Republic of China*, and the accurate Latin name was not mentioned in the literature.

**Table 3:** Flavanones and flavanonols identified from dandelion.

No.	Compounds	*Taraxacum* Species	Parts	References
107	5,7-dihydroxyflavanone	*Taraxacum mongolicum* Hand.-Mazz	whole herbs	[69]
108	naringenin	*Taraxacum officinale*	leaves	[81]
		*Taraxacum mongolicum*	leaves	[80]
		*Taraxacum mongolicum* Hand.-Mazz	whole herbs	[69]
		*Taraxacum extractum*	aerial parts	[70]
109	naringenin-7-*O*-glucoside	fermented *Taraxacum officinale*	whole herbs	[68]
110	hesperetin	*Taraxacum mongolicum* Hand.-Mazz	whole herbs	[31,33,43]
111	hesperidin	*Taraxacum mongolicum* Hand.-Mazz	whole herbs	[26,29,31,33,43,52]
		*Taraxacum mongolicum* Hand.-Mazz	aerial parts	[49]
112	hesperetin-7-glucuronide	*Taraxacum mongolicum* Hand.-Mazz	whole herbs	[52]
113	hesperetin-5′-*O*-*β*-rhamnoglucoside	*Taraxacum mongolicum* Hand.-Mazz	whole herbs	[52]
114	4′,5,7-trihydroxy-3′-methoxyflavanone	*Taraxacum mongolicum* Hand.-Mazz	aerial parts	[29]
115	liquiritigenin	fermented *Taraxacum officinale*	whole herbs	[68]
116	liquiritin	fermented *Taraxacum officinale*	whole herbs	[68]
117	farrerol	fermented *Taraxacum officinale*	whole herbs	[68]
118	garbanzol	*Taraxacum officinale*	whole herbs	[68]
119	toxifolin	*Taraxaci Herba*	roots	[54]
		*Taraxacum mongolicum* Hand.-Mazz	whole herbs	[73]
120	(2*R*,3*R*)-(+)-4′-*O*-methyldihydro-quercetin	*Neo-Taraxacum siphonanthum*	whole herbs	[60]
121	(2*R*,3*R*)-(+)-4′,7-di-*O*-methyldihydro-quercetin	*Neo-Taraxacum siphonanthum*	whole herbs	[60]
122	dihydromyricetin	Fermented *Taraxacum officinale*	whole herbs	[68]
123	silymarin	*Taraxaci Herba*	roots	[54]

Note: *Taraxaci Herba*: The common name of dandelion in the *Pharmacopoeia of the People’s Republic of China*, and the accurate Latin name was not mentioned in the literature.

**Table 4:** Anthocyanidins and flavan-3-ols identified from dandelion.

No.	Compounds	*Taraxacum* Species	Parts	References
124	cyanidin	*Taraxaci Herba*	roots	[82]
		fermented *Taraxacum officinale*	whole herbs	[68]
125	cyanidin-3-glucoside	*Taraxacum officinale*	leaves	[83]
		*Taraxacum brevicorniculatum*	leaves	[77]
126	delphinidin-3-*O*-glucoside	*Taraxacum officinale*	whole herbs	[68]
127	cyanidin-3-(6-malonyl)-glucoside (A-1)	*Taraxacum officinale*	leaves	[83]
128	cyanidin-3-(6-malonyl)-glucoside (A-2)	*Taraxacum officinale*	leaves	[83]
129	peonidin-3-(6-malonyl)-glucoside	*Taraxacum officinale*	leaves	[83]
130	catechin	*Taraxaci Herba*	extracts	[44]
131	(+)-catechin	*Taraxacum officinale*	whole herbs	[84]
132	(−)-epicatechin	*Taraxacum officinale*	whole herbs	[84]
133	(−)-epigallocatechin	*Taraxacum officinale*	whole herbs	[84]
134	(−)-epigallocatechingallate	*Taraxacum officinale*	whole herbs	[84]

Note: *Taraxaci Herba*: The common name of dandelion in the *Pharmacopoeia of the People’s Republic of China*, and the accurate Latin name was not mentioned in the literature.

**Table 5:** Chalcones, dihydrochalcones, isoflavones, xanthones and biflavonoids identified from dandelion.

No.	Compounds	*Taraxacum* Species	Parts	References
135	butein	*fermented Taraxacum officinale*	whole herbs	[68]
136	xanthium	*fermented Taraxacum officinale*	whole herbs	[68]
137	loureirin A	*Taraxacum kok-saghyz* Rodin	roots and leaves	[55]
138	isoliquiritigenin	*fermented Taraxacum officinale*	whole herbs	[68]
139	phloretin	*Taraxacum mongolicum* Hand.-Mazz	whole herbs	[73]
140	daidzein	*Taraxacum officinale*	whole herbs	[68]
141	2′-hydroxyxydaidzein	*fermented Taraxacum officinale*	whole herbs	[68]
142	genistein	*Taraxacum mongolicum* Hand.-Mazz	whole herbs	[69]
		*Taraxacum extractum*	aerial parts	[70]
143	glycitein	*Taraxacum mongolicum* Hand.-Mazz	whole herbs	[69]
144	tectorigenin	*Taraxacum kok-saghyz* Rodin	roots and leaves	[55]
		*Taraxacum extractum*	aerial parts	[70]
145	iristectorigenina	*Taraxacum kok-saghyz* Rodin	roots and leaves	[55]
146	pseudobaptigenin	*Taraxacum extractum*	aerial parts	[70]
147	formononetin	*Taraxacum extractum*	aerial parts	[70]
148	sophoricoside	*Taraxacum coreanum* Nakai	aerial parts	[79]
149	genistin	*Taraxacum extractum*	aerial parts	[70]
150	mangostenone B	*Taraxaci Herba*	not mentioned	[78]
151	philonotisflavone	*Taraxacum officinale* L.	fruits	[67]
152	luteolin-luteolin	*Taraxacum officinale* L.	fruits	[67]
153	luteolin-apigenin	*Taraxacum officinale* L.	fruits	[67]
154	luteolin-chrysoeriol	*Taraxacum officinale* L.	fruits	[67]
155	amentoflavone	*Taraxacum officinale*	whole herbs	[68]

Note: *Taraxaci Herba*: The common name of dandelion in the *Pharmacopoeia of the People’s Republic of China*, and the accurate Latin name was not mentioned in the literature.

**Table 6:** Methods used for identification or quantification of flavonoids from dandelion.

No.	Compounds	Methods	Columns	Mobile Phases	Flow Rate (mL/min)	Ionization Modes	References
1	**62**, **89**, **130**	HPLC	SCION-C_18_ (4.6 mm × 250 mm, 0.45 μm)	A: methanol, B: 0.2% formic acid solution	0.3~1.0	no mass spectrometry	[44]
2	**41**, **84**, **119**, **123**	UPLC-Q-Exactive-Orbitrap MS	Hypersil GOLD aQ (2.1 mm × 100 mm, 1.9 μm)	A: 0.1% formic acid-water, B: 0.1% formic acid-acetonitrile	0.3	ESI, positive and negative	[54]
3	**4**, **33**, **36**, **37**, **61**, **89**, **144**, **145**	UHPLC-Q/Orbitrap HRMS	Waters ACQUITY BEH C_18_ (2.1 mm × 100 mm, 1.7 μm)	A: 0.1% formic acid-water, B: 0.1% formic acid-acetonitrile	0.5	APCI, positive and negative	[55]
4	**8**, **31**, **73**, **91**, **95**, **99**, **100**	UHPLC-QTOF-MS/MS	not mentioned	A: 0.1% formic acid water, B: 0.1% formic acid: acetonitrile	0.8	ESI, negative	[66]
5	**4**, **5**, **18**, **21**, **22**, **25**, **44**, **45**, **151**, **152**, **153**, **154**	UHPLC-PDA-CAD-ESI-QTOF-MS/MS	HSS C_18_ (2.1 mm × 100 mm, 1.7 μm)	A: 0.1% formic acid water, B: 0.1% formic acid acetonitrile	0.4	ESI, positive and negative	[67]
6	**2**, **35**, **38**, **60**, **72**, **84**, **109**, **115**, **116**, **117**, **118**, **122**, **124**, **126**, **135**, **136**, **138**, **140**, **141**, **155**	LC-ESI-MS/MS	Waters ACQUITY UPLC HSS T3 C_18_ (2.1 mm × 100 mm, 1.8 μm)	A: 0.04% acetic acid in water, B: 0.04% acetic acid in acetonitrile	0.4	ESI, positive	[68]
7	**1**, **26**, **27**, **30**, **35**, **56**, **76**, **77**, **92**, **105**, **107**, **108**, **142**, **143**	HPLC-Q-TOF-MS	HPLC ODS C_18_ (4.6 mm × 250 mm, 5 μm)	A: acetonitrile, B: 0.1% formic acid water	1.0	ESI, negative	[69]
8	**3**, **5**, **11**, **29**, **30**, **44**, **63**, **78**, **84**, **92**, **108**, **142**, **144**, **146**, **147**, **149**	UHPLC-HRMS/MS	Accucore UHPLC Column C_18_ (2.1 mm × 150 mm, 2.6 μm)	A: ultrapure water containing 500 µL/L formic acid (pH 2.5), B: methanol with 500 µL/L formic acid	0.3	ESI, negative	[70]
9	**4**, **6**, **13**, **25**, **39**, **46**, **62**, **63**, **87**, **88**	HPLC-DAD-MS/MS	Agilent ZORBAX Eclipse Plus C_18_ (4.6 mm × 250 mm, 5 μm)	A: 0.1% formic acid water, B: methanol	1.0	negative	[71]
10	**16**, **37**, **44**, **47**, **48**, **56**, **57**, **58**, **68**, **71**, **92**, **93**, **94**, **98**, **119**, **139**	UPLC-MS/MS	ACQUITY UPLC HSS T3 C_18_ (2.1 mm × 100 mm, 1.8 μm)	A: 0.04% acetic acid water, B: 0.04% acetic acid acetonitrile	0.4	not mentioned	[73]
11	**103**, **150**	UPLC-QTOF MS	ACQUITY UPLC BEH C_18_ (2.1 mm × 100 mm, 1.7 μm)	A: 0.1% formic acid water, B: 0.1% formic acid: acetonitrile	0.4	ESI, positive and negative	[78]
12	**4**, **6**, **64**, **67**, **69**, **70**, **97**, **148**	UHPLC-ESI-MS	Waters Cortex T3 (2.1 mm × 150 mm, 1.6 μm)	A: 0.1% formic acid water, B: 0.1% formic acid: acetonitrile	0.25	ESI, positive	[79]
13	**125**, **127**, **128**, **129**	LC-ESI-HR-QTOF-MS	Zorbax Eclipse Plus C_18_ (2.1 mm × 50 mm, 1.8 μm)	A: 0.1% formic acid water, B: 0.1% formic acid acetonitrile	0.5	ESI, positive	[83]
14	**131**, **132**, **133**, **134**	HPLC	Symmetry C_18_ (4.6 mm × 250 mm, 5 μm)	A: 50 mM ammonium phosphate monobasic (NH_4_H_2_PO_4_), pH 2.6; B: 80:20 (*v*/*v*) acetonitrile/50 mM pH 2.6; C: 200 mM phosphoric acid (H_3_PO_4_), pH 1.5	1.0	no mass spectrometry	[84]
15	**4**, **62**, **64**, **67**, **69**, **70**	HPLC-ESI-MS/MS	Zorbax SB C_18_ (4.6 mm × 250 mm, 5 μm)	A: methanol, B: 0.1% acetic acid solution	1.0	ESI, negative	[85]

Note: HPLC: high-performance liquid chromatography; UPLC-Q-Exactive-Orbitrap MS: ultra-high-performance liquid chromatography-tandem hybrid quadrupole-electrostatic field orbitrap high resolution mass spectrometry; UHPLC-Q/Orbitrap HRMS: ultra-high-performance liquid chromatography coupled with quadrupole/Orbitrap high resolution mass spectrometry; UHPLC-QTOF-MS/MS: ultra-high-performance liquid chromatography coupled with quadrupole time of flight mass spectrometry; UHPLC-PDA-CAD-ESI-QTOF-MS/MS: ultra-high-performance liquid chromatography coupled with diode array detector, corona-charged aerosol detector, and quadrupole time of flight mass spectrometry; LC-ESI-MS/MS: liquid chromatography electrospray ionization mass spectrometry; HPLC-Q-TOF-MS: ultra-high-performance liquid chromatography coupled with quadrupole time of flight mass spectrometry; UHPLC-HRMS/MS: ultra-high-performance liquid chromatography with high-resolution mass spectrometry; HPLC-DAD-MS/MS: high-performance liquid chromatography diode array detector mass spectroscopy; UPLC-MS/MS: ultra-performance liquid chromatography-tandem mass spectrometry; UPLC-QTOF MS: ultra-high-performance liquid chromatography coupled with quadrupole time of flight mass spectrometry; UHPLC-ESI-MS: ultra-liquid chromatography electrospray ionization mass spectrometry; LC-ESI-HR-QTOF-MS: liquid chromatography electrospray ionization with high-resolution quadrupole time of flight mass spectrometry; HPLC-ESI-MS/MS: high-performance liquid chromatography electrospray ionization mass spectrometry; ESI: electrospray ionization; APCI: pressure chemical ionization source.

## 4. Antioxidant Activities and Mechanisms

**Figure 6:**
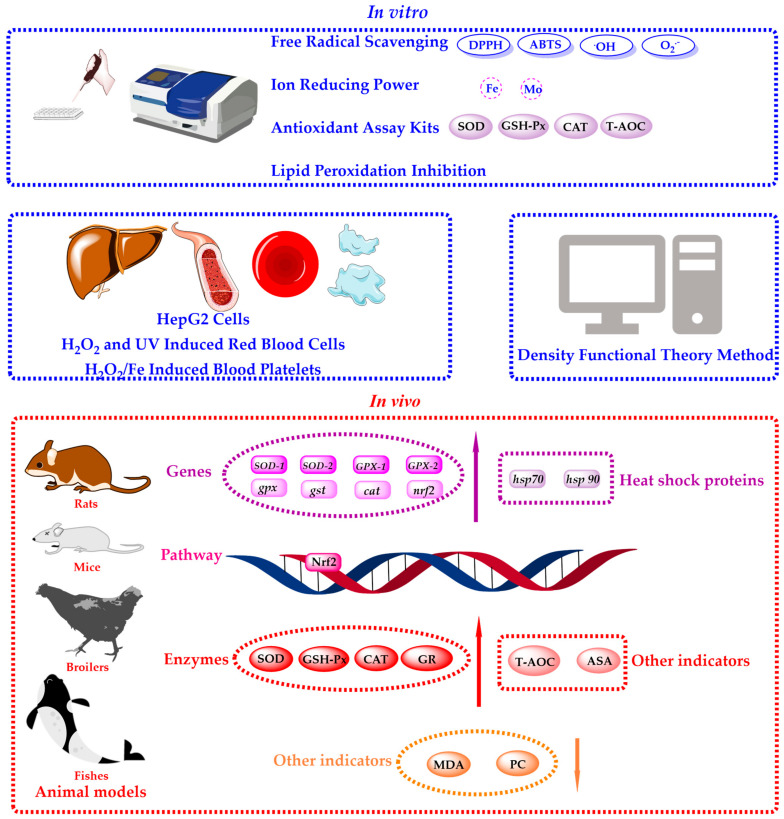
In vitro and in vivo antioxidant activities of flavonoids derived from dandelion. Note: DPPH: 1,1-diphenyl-2-picrylhydrazyl; ABTS: 2,2′-azinobis-(3-ethylbenzthiazoline)-6-sulfonic acid; ·OH: hydroxyl radical; O_2_^•−^: superoxide anion radical; T-AOC: total antioxidant capacity; SOD: superoxide dismutase; GSH-Px: glutathione peroxidase; CAT: catalase; GR: glutathione reductase; MDA: malondialdehyde; ASA: ascorbic acid; PC: protein carbonyl.

Numerous reports show that the in vitro and in vivo antioxidant activities of flavonoids in dandelion mainly relate to flavonoid-containing dandelion extracts, and there are only a few reports on flavonoid monomer compounds [52,87,88]. As shown in Figure 6, antioxidant activities’ research primarily focused on in vitro and in vivo aspects.

### 4.1. In Vitro Antioxidant Activities and Mechanisms

In vitro antioxidant studies are important methods for studying the antioxidant activities of dandelion flavonoids due to their low cost and ease of operation. Commonly used methods include free radical scavenging assays, ion-reducing power determinations, antioxidant assay kits, lipid peroxidation inhibition, cell experiments, and density functional theory (DFT). Free radical scavenging assays include 1,1-diphenyl-2-picrylhydrazyl (DPPH) radical scavenging assay [52], 2,2′-azinobis-(3-ethylbenzthiazoline)-6-sulfonic acid (ABTS) radical scavenging assay [15], hydroxyl radical scavenging assay and superoxide anion radical scavenging assay [89]. Ion-reducing power determinations include the reducing power assay [15] and the total ferric-reducing antioxidant power (FRAP) assay [90]. Additionally, antioxidant assay kits can be employed for the quick assessment of various antioxidant properties, including superoxide dismutase (SOD), glutathione peroxidase (GSH-Px), catalase (CAT), and total antioxidant capacity (T-AOC) [88]. In addition, lipid peroxidation inhibition capacities are also important indicators of antioxidant activities, which can prevent cells from being damaged by lipid peroxyl radicals [91]. Moreover, cell models, which more closely mimic the internal environment of organisms, are widely used to study the antioxidant activities of dandelion flavonoids [92,93]. At the same time, quantum chemical density functional theory methods can also evaluate the antioxidant activities of dandelion flavonoids and explore the structure–activity relationship between dandelion flavonoids and antioxidant activities [52]. As shown in Table 7, various in vitro antioxidant methods were typically used for comprehensively evaluating the antioxidant activities of dandelion flavonoids.

#### 4.1.1. Free Radical Scavenging

During normal metabolism, excessive free radicals are produced due to factors, such as self-activities, emotional fluctuations, drugs, alcohol, and radiation. These free radicals can exceed the body’s scavenging capacity, leading to a dynamic imbalance between their production and elimination, causing oxidative damage, which is closely related to various diseases and aging [16,23]. Therefore, scavenging unnecessary free radicals is absolutely essential for the treatment of related diseases and anti-aging. In addition, the generation of large amounts of free radicals can cause food, feed, and cosmetics to spoil during the procedures of production, storage, transportation, and use, affecting their normal edible value and consumption function [88,94]. Hence, adding safe natural antioxidants to food, feed, or cosmetics to prevent lipid peroxidation is of great significance. Dandelion flavonoids, such as luteolin (**4**) and quercetin (**62**), can act as electron donors, effectively reducing and scavenging free radicals, demonstrating significant antioxidant activities [95].

##### DPPH Radical Scavenging

The DPPH radical scavenging activity is relatively extensive in the in vitro antioxidant activities of dandelion flavonoids. From stems and leaves to roots, different parts of dandelion possess various antioxidant potentials, whilst stems and leaves’ total flavonoids extracts exhibit almost 2.25-times stronger DPPH radical scavenging capacity than roots’ total flavonoids extract. In addition, it was found that their concentrations for 50% of maximal effect (EC_50_) values were 54.88 μg/mL and 123.50 μg/mL, respectively [96]. There is also a study showing that the DPPH scavenging rate of dandelion after solid-state fermentation with probiotics was 90.7%, which increased by 1.28-times compared with before fermentation. Simultaneously, the flavonoid content after fermentation increased by 2.74-times compared with before fermentation. Therefore, the study provided a new way to enhance the antioxidant capacity of dandelion [97]. There was a study that used the DPPH assay to study the in vitro antioxidant activities of various fractions of dandelion flower extract. It was found that dandelion flowers with different polar solvent fractions have varying degrees of antioxidant activities, with the order of effects as follows: ethyl acetate fraction > *n*-butanol fraction > aqueous fraction > petroleum ether fraction. The antioxidant activities were positively correlated with the contents of flavonoids [98]. A flavonoid extract of dandelion was reported to possess beneficial effects by scavenging DPPH radicals. In addition, the DPPH radical scavenging rate was 50.11% when the concentration of the flavonoid extract was 0.7 mg/mL. Meanwhile, the scavenging ability of dandelion flavonoid extract on DPPH free radicals was positively correlated with the concentration of the flavonoid extract [99]. Similarly, the total flavonoids extracted by ethanol from dandelion were reported to have good DPPH radical scavenging activity, with a concentration of 50% maximal inhibition (IC_50_) value of 0.8492 mg/mL [100]. According to the literature report, light intensity had an impact on the DPPH radical scavenging activities of *T. mongolicum* Hand.-Mazz. As the light intensity decreased, the alcohol extract of *T. mongolicum* Hand.-Mazz showed a trend of first increasing and then decreasing in its ability to scavenge DPPH free radicals. The DPPH scavenging ability was highest when treated with 80% light transmittance. The 20% light transmittance treatment had the lowest DPPH clearance rate, with no significant difference compared to the 40% light transmittance treatment. A correlation analysis suggested that the scavenging ability of DPPH free radicals was positively correlated with the contents of active ingredients, such as total flavonoids, total triterpenoids, total phenols, and total cholines [89]. It was also reported that 95% ethanol and subsequent water extracts of *T. officinale* L. Weber ex F.H. Wigg. roots collected from Northern and South Bulgaria locations possessed good antioxidant activities. In particular, subsequent water extracts from the Parvomay location demonstrated significant DPPH scavenging capacity, with a Trolox equivalent concentration 83.1 ± 3.2 mg TE/g dw (dry weight) [90]. It was found that *T. Lambinonii* was rich in phenolics and flavonoids, and possessed significant DPPH free radical scavenging activity, with an IC_50_ value of 0.083 ± 0.006 mg/mL when using the DPPH assay to study the antioxidant activities of methanol flower extracts from different species of dandelion, such as *T. obovatum* (Willd.) DC., *T. marginellum* H. Lindb., *T. hispanicum* H. Lindb., *T. lambinonii* Soest and *T. lacistrum* Sahlin [93]. In a certain study, it was found that the EC_50_ value of *T. officinale (L.)* Weber leaves’ extract for DPPH free radical scavenging was 207 ± 0.84 µg/mL, which showed potential in protection against sodium dichromate-induced hepatotoxicity [87]. The master’s thesis of Longfei Gao studied the effects of *T. mongolicum* flavonoids on the antioxidant activities of *Caragana koraiensis* silage by using the DPPH assay. It was found that after 60 days of *C. koraiensis* silage, different added amounts of *T. mongolicum* flavonoids significantly improved its DPPH free radical scavenging abilities. When the amount of *T. mongolicum* flavonoids added was 2.0%, the antioxidant activity of *C. koraiensis* silage was noteworthy [14]. In a study by Sun et al., total flavonoids enriched by ultrasonic-assisted extraction presented good DPPH radical scavenging capacity, with an IC_50_ value of 180.11 ± 7.85 μg/mL [101]. Similarly, the methanolic extract from *T. officinale* (IC_50_: 32.80 ± 9.66 μg/mL) revealed nearly 1.29- and 1.83-times higher DPPH radical scavenging activity than the acetone (IC_50_: 42.63 ± 5.55 μg/mL) and *n*-hexane extracts (IC_50_: 60.0 ± 8.37 μg/mL) [102]. A report indicated that the DPPH radical 50% scavenging concentrations of water extract from *T. officinale* and water extract formula derived from *Maydis stigma*, *Nelumbo nucifera* Gaertn and *T. officinale* were 0.41 ± 0.02 and 0.39 ± 0.02 mg/mL, respectively [103]. Notably, the extract of leaves from *T. officinale* showed nearly 3.62-times higher antioxidant potential compared to the extract of roots for DPPH radical scavenging capacity, with EC_50_ values of 0.37 and 1.34 mg/mL, respectively [104].

A study showed that the new type of flavonoid compound hesperetin-5′-*O*-*β*-rhamnoglucoside (**113**) had significant antioxidant activity, with an IC_50_ value of 8.72 mg/L, by using the DPPH assay to detect the antioxidant activity of flavonoids isolated from *T. mongolicum* Hand.-Mazz. The results showed that the DPPH radical scavenging activities’ sequence of these flavonoids was as follows: quercetin (**62**) (IC_50_ = 8.07 ± 0.67 mg/L) > hesperetin-5′-*O*-*β*-rhamnoglucoside (**113**) (IC_50_ = 8.72 ± 0.88 mg/L) > kaempferol-3-glucoside (**90**) (IC_50_ = 13.49 ± 1.02 mg/L) > baicalin (**35**) (IC_50_ = 15.5 ± 0.98 mg/L) > hesperetin-7-glucuronide (**112**) (IC_50_ = 22.1 ± 0.76 mg/L) > hyperseroside (**101**) IC_50_ = 31.39 ± 0.65 mg/L) > rutin (**84**) (IC_50_ = 31.54 ± 0.79 mg/L) [52]. Moreover, 32 compounds, including 15 flavonoids, were isolated and separated from *T. mongolicum* via high-performance liquid chromatography–diode array detection–radical scavenging detection–electrospray ionization mass spectrometry and nuclear magnetic resonance experiments. Among them, isoetin-7-*O*-*β*-D-glucosyranosyl-2′-*O*-*α*-D-glucopyranoside (**53**), isoetin-7-*O*-*β*-D-glucosyranosyl-2′-*O*-*β*-D-xyloypyranoside (**55**), and quercetin (**62**) showed strong DPPH radical scavenging activities, with IC_50_ values of 21.57 ± 2.53 µmol/L, 19.76 ± 2.83 µmol/L and 5.53 ± 0.76 µmol/L, respectively [29].

##### ABTS Radical Scavenging

From stems and leaves to roots, different parts of dandelion possess various antioxidant potentials, whilst stems and leaves’ total flavonoids extract exhibited almost 2.44-times stronger ABTS radical scavenging capacity than roots’ total flavonoids extract. In addition, it was found that their EC_50_ values were 229.41 μg/mL and 559.07 μg/mL, respectively [96]. Obviously, this result was consistent with the result measured by the DPPH assay. According to the literature report, light intensity had an impact on the ABTS radical scavenging activities of *T. mongolicum* Hand.-Mazz. As the light intensity decreased, the ability of dandelion alcohol extracts to scavenge ABTS free radicals in the natural light group was not significantly different from that of the 80% and 60% light transmittance treatments, with higher ABTS radical scavenging ability at 60% light transmittance. A correlation analysis suggested that the scavenging ability of ABTS free radicals was positively correlated with the contents of total phenols [89]. A study used the ABTS free radical scavenging rate as the detection index, with quercetin (**62**), luteolin (**4**), and rutin (**84**) as the research objects, to investigate the protective effects of *β*-lactoglobulin (*β*-LG) on the antioxidant activities of three flavonoids under different temperatures (25 °C, 65 °C, 75 °C, 90 °C) and natural light conditions on sunny summer days. It was found that the antioxidant activity of quercetin alone significantly decreased at 65 °C, 75 °C, and 90 °C, while the antioxidant activity of the *β*-LG-quercetin complex was significantly higher than that of quercetin alone at 25 °C, 65 °C, 75 °C, and 90 °C. Therefore, *β*-LG slowed down the decrease in the antioxidant activity of quercetin. Luteolin (**4**) showed no significant changes in antioxidant activity within a range of 25 °C, 65 °C, 75 °C, and 90 °C, while *β*-LG-luteolin showed the greatest decrease compared with luteolin (**4**) alone at 75 °C. The antioxidant activity of rutin (**84**) increased as the temperature increased, and the antioxidant activity of *β*-LG-rutin significantly decreased at 75 °C compared with rutin (**84**) alone [15]. In a study by Sun et al., total flavonoids enriched by ultrasonic-assisted extraction presented good ABTS radical scavenging capacity, with an IC_50_ value of 10.18 ± 1.07 μg/mL [101]. Furthermore, one report indicated that the Trolox equivalent antioxidant capacity of a water extract from *T. officinale* and water extract formula derived from *M. stigma*, *N. nucifera* Gaertn and *T. officinale* was 276.7 ± 45.8 and 454.2 ± 15.7 mM/mg, respectively [103]. Notably, the extract of leaves from *T. officinale* showed almost 2.38-times higher antioxidant potential compared to the extract of roots for ABTS radical scavenging capacity, with 407.5 ± 0.14 and 171.5 ± 1.01 µM TE/mg of extract, respectively [104].

##### Hydroxyl Radical Scavenging

The study also used the hydroxyl radical scavenging assay to study the in vitro antioxidant activities of various fractions of dandelion flower extract. It was found that dandelion flowers with different polar solvent fractions have varying degrees of antioxidant activities, with the order of effects as follows: ethyl acetate fraction > *n*-butanol fraction > aqueous fraction > petroleum ether fraction. The antioxidant activities were positively correlated with the contents of flavonoids [98]. Obviously, this result was consistent with the result measured by the DPPH assay. Additionally, the flavonoid extract of dandelion was reported to possess beneficial effects by scavenging hydroxyl radicals, which was similar to the effect of scavenging DPPH radicals. Namely, the hydroxyl radical scavenging rate was 59.00% when the concentration of flavonoid extract was 0.7 mg/mL. Meanwhile, the scavenging ability of dandelion flavonoid extract on hydroxyl free radicals was positively correlated with the concentration of the flavonoid extract [99]. Similarly, the total flavonoids extracted by ethanol from dandelion were reported to have good hydroxyl radical scavenging activity, with an IC_50_ value of 1.0717 mg/mL [100]. According to the literature report, light intensity had an impact on the hydroxyl radical scavenging activities of *T. mongolicum* Hand.-Mazz. The hydroxyl radical scavenging ability of dandelion alcohol extracts first increased and then decreased as the light intensity decreased, with the highest scavenging ability at a 60% light transmittance treatment, significantly higher than other treatments [89]. Moreover, a study indicated that the hydroxyl radical scavenging activity of *T. mongolicum* Hand.-Mazz total flavonoids was stronger than that of rutin (**84**), quercetin (**62**), and positive control vitamin E at concentrations of 1 mg/mL, 4 mg/mL, and 16 mg/mL, indicating strong reactive oxygen species (ROS) scavenging activity [92].

##### Superoxide Anion Radical Scavenging

The study also used the superoxide anion radical scavenging assay to study the in vitro antioxidant activities of various fractions of dandelion flower extract. It was found that dandelion flowers with different polar solvent fractions have varying degrees of antioxidant activities, with the order of effects as follows: ethyl acetate fraction > *n*-butanol fraction > aqueous fraction > petroleum ether fraction. The antioxidant activities were positively correlated with the contents of flavonoids [98]. Obviously, this result was consistent with the result measured by the DPPH assay and the hydroxyl radical scavenging assay. However, according to the literature report, light intensity had no impact on the superoxide anion radical scavenging activities of *T. mongolicum* Hand.-Mazz [89]. It was reported that *T. obovatum* had significant superoxide anion radical scavenging activity, with an IC_50_ value of 0.199 ± 0.015 mg/mL when using the superoxide anion radical scavenging assay to study the antioxidant activities of methanol flower extracts from different species of dandelion, such as *T. obovatum* (Willd.) DC., *T. marginellum* H. Lindb., *T. hispanicum* H. Lindb., *T. lambinonii* Soest and *T. lacistrum* Sahlin [93]. Meanwhile, a study indicated that the superoxide anion scavenging activity of *T. mongolicum* Hand.-Mazz total flavonoids was almost 1.54- and 2.26-times stronger than that of rutin (**28**) and quercetin (**62**), indicating strong ROS scavenging activity [92]. It was found that 1 mg of *T. mongolicum* Hand.-Mazz flavonoids extract was equivalent to 21.41 SOD vitality units, while 1 mg of rutin (**84**) was equivalent to 13.89 SOD vitality units, indicating strong in vitro superoxide anion radical scavenging abilities for both *T. mongolicum* Hand.-Mazz flavonoids extract and rutin (**84**) [105]. As reported, the hydroalcoholic extract of *T. officinale* exhibited notable radical scavenging ability at 830.78 µM TE/g, dw [91].

#### 4.1.2. Ion-Reducing Power Determination

One study used iron ion-reducing power as the detection index, with quercetin (**62**), luteolin (**4**), and rutin (**84**) as the research objects, to investigate the protective effects of *β*-LG on the antioxidant activities of three flavonoids under different temperatures (25 °C, 65 °C, 75 °C, 90 °C) and natural light conditions on sunny summer days. The results were basically consistent with the results of the ABTS assay mentioned above [15]. Moreover, 95% ethanol and subsequent water extracts of *T. officinale* L. Weber ex F.H. Wigg. roots collected from Northern and South Bulgaria locations were proved to possess good antioxidant activities via FRAP assay. In particular, subsequent water extracts from the Parvomay location demonstrated significant iron ion-reducing power, with a Trolox equivalent concentration of 46.9 ± 1.3 mg TE/g dw [90]. Wasim Akhtar et al. found that the methanolic extract from *T. officinale* possessed good reducing potential and total antioxidant capacity (TAC), with values of 0.53 ± 0.02 mg/g and 19.42 ± 0.97 mg/g, respectively [102]. A report indicated that the ascorbic acid equivalent antioxidant capacities of a water extract from *T. officinale* and water extract formula derived from *M. stigma*, *N. nucifera* Gaertn and *T. officinale* were 536.1 ± 49.0 and 485.1 ± 50.9 mM/mg, respectively [103]. Notably, the extract of leaves from *T. officinale* showed almost 3.9-times higher antioxidant potential compared to the extract of roots for ion-reducing capacity, with 156 ± 5.28 compared to 40 ± 0.3 µg VC/mg extract, respectively [91].

#### 4.1.3. Antioxidant Assay Kits

The master’s thesis of Longfei Gao studied the effects of *T. mongolicum* flavonoids on the antioxidant activity of *C. korshinskii* silage by using antioxidant assay kits produced by Nanjing Jiancheng Bioengineering Institute. It was found that T-AOC first decreased and then stabilized; GSH-Px activity first increased and then stabilized; and SOD activity did not fluctuate much throughout the silage process. After 60 days of silage, different amounts of *T. mongolicum* flavonoids significantly increased the T-AOC and GSH-Px activities of *C. korshinskii* silage, with significant antioxidant activity at a *T. mongolicum* flavonoids addition of 2.0% [14]. This result, as well as the result of the DPPH assay, indicated that different amounts of *T. mongolicum* flavonoids could enhance the antioxidant activities of *C. korshinskii* silage feed. The antioxidant activity of *C. korshinskii* silage was significant when the *T. mongolicum* flavonoids addition was 2.0%. Similarly, total flavonoids from *T. mongolicum* Hand.-Mazz. used as additives were proved to promote the antioxidant activities of *C. korshinskii* silage by testing with antioxidant assay kits produced by Nanjing Jiancheng Bioengineering Institute. It was indicated that the T-AOC, SOD, and CAT activities of the 2% *T. mongolicum* Hand.-Mazz. total flavonoids group were higher than those of the group without *T. mongolicum* Hand.-Mazz. total flavonoids and the 1% *T. mongolicum* Hand.-Mazz. total flavonoids group. The T-AOC and CAT activities of the 1% *T. mongolicum* Hand.-Mazz. total flavonoids group were higher than those of the group without *T. mongolicum* Hand.-Mazz. total flavonoids, and the GSH-Px activities of both the 1% and 2% *T. mongolicum* Hand.-Mazz. total flavonoids groups were higher than those of the group without *T. mongolicum* Hand.-Mazz. total flavonoids [88]. Therefore, the addition of *T. mongolicum* Hand.-Mazz. total flavonoids would contribute to improving the antioxidant activities of *C. korshinskii* silage, similar to the findings of Longfei Gao.

#### 4.1.4. Lipid Peroxidation Inhibition

In a study by Sun et al., total flavonoids enriched from *T. officinale* by ultrasonic-assisted extraction presented better lipid peroxidation inhibition capacity compared with vitamin C in the *β*-carotene bleaching assay (CB), with inhibition rates of 55.78% and 4.30%, respectively. However, it was less efficient than BHT in both CB and ferrothiocyanate assays (FTC) [101]. As reported, the hydroalcoholic extract of *T. officinale* was effective in protecting liposomes from lipid peroxidation, with an IC_50_ value of 98.49 ± 6.67 µg/mL [91].

#### 4.1.5. Cell Experiments

It was found that the antioxidant activities of the methanol extracts from different species of dandelion were dose-dependent by testing ROS in HepG2 cells. In addition, each species of dandelion had a different response pattern. *T. obovatum*, *T. hispanicum* and *T. lacistrum* could not reverse the increase in ROS concentration caused by the oxidant (H_2_O_2_) [93]. The antioxidant activities of total flavonoids in *T. mongolicum* Hand.-Mazz. were proved to be almost 1.04-, 1.12-, and 1.00-times stronger than that of rutin (**28**), quercetin (**62**), and vitamin E at a concentration of 4 mg/L by H_2_O_2_-induced red blood cell hemolysis experiments. Furthermore, the antioxidant activities of total flavonoids in *T. mongolicum* Hand.-Mazz. were proved to be slightly weaker than that of vitamin E by ultraviolet (UV)-induced red blood cell hemolysis experiments. Overall, *T. mongolicum* Hand.-Mazz. flavonoids showed strong ROS scavenging activities [92]. A previous study suggested that leaves and petals 50% methanol fractions of *T. officinale* could be a new source of natural compounds, showing cooperative activities: antioxidant, anti-platelet and anticoagulant, beneficial in the prevention and treatment of cardiovascular diseases, which are often associated with changes in hemostasis and oxidative stress [106].

#### 4.1.6. Density Functional Theory Method

The density functional theory method can be used to study the relationships between the structures and antioxidant activities of flavonoids isolated from dandelions. It was found that the antioxidant activities were weak when the phenolic hydroxyl groups of the flavonoids were in the A-ring. The antioxidant activities were high when the phenolic hydroxyl groups were in the B-ring. Ortho-substituents in the B-ring were essential antioxidant groups for flavonoids, especially when they were substituted with phenolic hydroxyl groups. The antioxidant activities of 3-OH substitution in the C-ring were particularly important, and 3-OH glycosylations in the C-ring were not conducive to exerting antioxidant activities; the stronger the glycosylations, the worse the antioxidant activities. Therefore, the antioxidant activities were in the order of quercetin (**62**) (IC_50_: 8.07 ± 0.67 mg/L) > hesperetin-5′-*O*-*β*-rhamnoglucoside (**49**) (IC_50_: 8.72 ± 0.88 mg/L) > hesperetin-7-glucuronide (**48**) (IC_50_: 13.49 ± 1.02 mg/L). The spatial hindrance of large glycoside groups mainly played a role in shielding or hindering the 3,4-OH of the B-ring, resulting in reduced antioxidant activities. Through DFT calculations, the molecular orbital energy levels of flavonoids with strong antioxidant activities and the hydrogen extraction enthalpy of B-ring substituents were re-determined. Theoretical results were consistent with experimental results, proving that hesperetin-5′-*O*-*β*-rhamnoglucoside (**49**) had strong antioxidant activities, mainly due to the combined effects of different substituents in the B-ring and the enhanced hydrogen supply capacity [52].

**Table 7:** In vitro antioxidant activities of flavonoids extracted from dandelion.

No.	Extracts/Compounds	Sources	Assays	Results	References
1	Dandelion flavonoids	Research office of natural drug research center, school of pharmacy, Jilin University *	DPPH, ABTS	Both total flavonoids from dandelion stems and leaves showed good antioxidant capacity with EC_50_ values of 54.88 μg/mL and 123.50 μg/mL for DPPH, 229.41 μg/mL and 559.07 μg/mL for ABTS.	[96]
2	Probiotic synergistic dandelion fermentation	*Taraxacum mongolicum* Hand.-Mazz.	DPPH	The DPPH clearance rate after fermentation was 90.7%, which was 1.28 times higher than before fermentation.	[97]
3	Dandelion flavonoids	Shanxi Guanchen Biotechnology Co., Ltd. (Xi’an, China) *	DPPH	After 60 days of silage, different amounts of dandelion flavonoids significantly increased the DPPH free radical scavenging ability of *Caragana korshinshii*. The antioxidant activity of *Caragana korshinshii* silage was the best when the addition of dandelion flavonoids was 2.0%.	[14]
4	Different fractions of dandelion flowers extracts	*Taraxacum mongolicum* Hand.-Mazz.	DPPH, ·OH, O_2_^•−^	Different fractions of dandelion flower extracts had different antioxidant effects, and the order was as follows: ethyl acetate fraction > *n*-butanol fraction > water fraction > petroleum fraction.	[98]
5	Dandelion total flavonoids	*Taraxaci Herba*	DPPH, ·OH	When the concentration of flavonoids extract was 0.7 mg/mL, the clearance rates of DPPH and ·OH were 50.11% and 59.00%, respectively.	[99]
6	Dandelion total flavonoids	*Taraxacum mongolicum* Hand.-Mazz.	DPPH, ·OH	The IC_50_ value of DPPH free radical and ·OH scavenging rate was 0.8492 mg/mL and 1.0717 mg/mL, respectively.	[100]
7	Dandelion total flavonoids	*Taraxacum mongolicum* Hand.-Mazz.	DPPH, ABTS, ·OH, O_2_^•−^	The DPPH radical scavenging ability of dandelion alcohol extract showed a trend of first increasing and then decreasing as the light intensity decreases. The scavenging ability was highest in the 80% transmittance treatment, and lowest in the 20% transmittance treatment, with no significant difference from the 40% transmittance treatment. There was no significant difference in the ability of scavenging ABTS free radicals between the natural light group and the 80% and 60% light transmittance treatments. The ability of scavenging ABTS free radicals was high under 60% light transmittance treatment. The ability of scavenging ·OH showed a trend of first increasing and then decreasing. The ability of scavenging ·OH was highest in the 60% transmittance treatment. And the ability of scavenging ·OH in the 60% transmittance treatment, which was significantly higher than other treatments. There was no significant difference in the ability of scavenging O_2_^•−^ among different transmittance treatments.	[89]
8	95% Methanol extracts from dandelion different plant parts (total flavonoids)	*Taraxacum officinale* F. H. Wigg.	DPPH	The antioxidant activities of the methanol extracts from all the plant parts dose-dependently increased. DPPH free radical scavenging activity was highest in flower extracts (IC_50_ = 624.3 mg/kg), and followed by leaves, roots, and stalks extracts.	[4,107]
9	95% Ethanol and subsequent water extracts from dandelion roots (total flavonoids)	*Taraxacum officinale* L. Weber ex F.H. Wigg.	DPPH, FRAP	The subsequent water extracts of roots from the Parvomay location demonstrated the highest antioxidant activity (DPPH, 83.1 ± 3.2 mg TE/g dw; FRAP, 46.9 ± 1.3 mg TE/g dw), while water extracts from Plovdiv location showed high activity defined only by FRAP assay: 52.9 ± 0.3 mg TE/g dw.	[4,90]
10	80% Methanol extracts (total flavonoids)	*Taraxacum obovatum* (Willd.) DC., *Taraxacum marginellum* H. Lindb., *Taraxacum hispanicum* H. Lindb., *Taraxacum lambinonii* Soest and *Taraxacum lacistrum* Sahlin	DPPH, O_2_^•−^	*Taraxacum lambinoni* had the highest total phenolic and flavonoid contents, and the strongest DPPH free radical scavenging activity, with an IC_50_ value of 0.083 ± 0.006 mg/mL; *Taraxacum obovatum* had the strongest scavenging activity for O_2_^•−^, with an IC_50_ value of 0.199 ± 0.015 mg/mL.	[4,93]
11	Dandelion leaf extract (total flavonoids)	*Taraxacum officinale* Weber	DPPH	The EC_50_ value for DPPH radical scavenging was 207 ± 0.84 µg/mL.	[87]
12	Dandelion flavonoids, rutin, quercetin	*Taraxacum mongolicum* Hand.-Mazz.	O_2_^•−^, ·OH	The activities of dandelion total flavonoids in scavenging O_2_^•−^ and ·OH were stronger than that of rutin, quercetin, and positive control vitamin E. Dandelion flavonoids had strong activity in scavenging ROS.	[92]
13	Dandelion total flavonoids, rutin	*Taraxacum mongolicum* Hand.-Mazz.	O_2_^•−^	1 mg flavonoid extract was equivalent to 21.41 SOD active units, and 1 mg rutin was equivalent to 13.89 SOD active units. Both dandelion flavonoid extracts and rutin had strong ability to scavenge O_2_^•−^ in vitro.	[105]
14	Hesperetin-5′-*O*-*β*-rhamnoglucoside, hesperetin-7-glucuronide, kaempferol-3-glucoside, baicalein, hyperseroside	*Taraxacum mongolicum* Hand.-Mazz.	DPPH, DFT	The IC_50_ value of DPPH scavenging activity of hesperetin-5′-*O*-*β*-rhamnoglucoside was 8.72 mg/L, DPPH radical scavenging activity sequence: quercetin (8.07 ± 0.67 mg/L) > hesperetin-5′-O-*β*-rhamnoglucoside (8.72 ± 0.88 mg/L) > kaempferol-3-glucoside (13.49 ± 1.02 mg/L) > baicalein (15.5 ± 0.98 mg/L) > hesperetin-7-glucuronide (22.1 ± 0.76 mg/L) > hysperoside (31.39 ± 0.65 mg/L) > rutin (31.54 ± 0.79 mg/L).	[52]
15	Isoetin-7-O-*β*-D-glucopyranosyl-2′-O-*α*-D-glucopyranoside, quercetin, isoetin-7-O-*β*-D-glucopyranosyl- 2′-O-*β*-D-xyloypyranoside	*Taraxacum mongolicum* Hand.-Mazz.	DPPH	The IC_50_ values were 21.57 ± 2.53 µmol/L, 5.53 ± 0.76, and 19.76 ± 2.83 µmol/L, respectively.	[29]
16	Quercetin, luteolin, rutin	Shanghai Yuanye Biotechnology Co., Ltd. (Shanghai, China) *	ABTS, reducing power	The antioxidant activities of quercetin alone significantly decreased at 65 °C, 75 °C, and 90 °C, while the antioxidant activities of *β*-LG-quercetin complex was significantly higher than that of quercetin alone at 25 °C, 65 °C, 75 °C, and 90 °C. Therefore, *β*-LG slowed down the decrease of antioxidant activities of quercetin. Luteolin showed no significant changes in antioxidant activities within the range of 25 °C, 65 °C, 75 °C, and 90 °C, while *β*-LG-luteolin showed the greatest decrease compared with luteolin alone at 75 °C. The antioxidant activities of rutin increased as the temperature increasing, and the antioxidant activities of *β*-LG-rutin significantly decreased at 75 °C compared with rutin alone.	[15]
17	Dandelion flavonoids	Shanxi Guanchen Biotechnology Co., Ltd. *	Antioxidant assasy kits provided by Nanjing Jiancheng Bioengineering Institute	Compared with 0% dandelion flavonoids addition group, different amounts of dandelion flavonoids could improve the antioxidant activity of *C.* silage. During the whole silage process, T-AOC decreased first and then stabilized. The activity of GSH-Px increased first and then stabilized. SOD activity didn’t change much during the whole silage process. After 60 days of silage, different amounts of dandelion flavonoids significantly increased the T-AOC and GSH-Px activity of *Caragana korshinshii* silage. The T-AOC of 2.0% dandelion flavonoids addition group was the highest (2.90 mmol/g), and the SOD activity of 2.0% dandelion flavonoids addition group was significantly higher than that of 0% dandelion flavonoids addition group. The antioxidant of *Caragana korshinshii* silage was the best when the addition of dandelion flavonoids was 2.0%.	[14]
18	Dandelion total flavonoids	Shanxi Guanchen Biotechnology Co., Ltd. *	Antioxidant assay kits provided by Nanjing Jiancheng Bioengineering Institute (Nanjing, China)	After 60 days of *Caragana korshinshii* silage, the T-AOC, SOD, and CAT activities of 2% dandelion flavonoids addition group were higher than that of 0% dandelion flavonoids addition group and 1% dandelion flavonoids addition group, and the T-AOC and CAT activities of 1% dandelion flavonoids addition group were higher than that of 0% dandelion flavonoids addition group. The GSH-Px activities of 1% dandelion flavonoids addition group and 2% dandelion flavonoids addition group were higher than that of 0% dandelion flavonoids addition group.	[88]
19	80% Methanol extracts (total flavonoids)	*Taraxacum obovatum* (Willd.) DC., *Taraxacum marginellum* H. Lindb., *Taraxacum hispanicum* H. Lindb., *Taraxacum lambinonii* Soest and *Taraxacum lacistrum* Sahlin	ROS measurement in HepG2 cells	The antioxidant activities of 5 dandelion extracts were dose-dependent. The response patterns were different for each species, three of them being unable to reverse the ROS concentration increase generated by the oxidizing agent (H_2_O_2_): *Taraxacum obovatum*, *Taraxacum hispanicum* and *Taraxacum lacistrum*. *Taraxacum marginellum* was the most efficient extract reducing intracellular ROS levels.	[4,93]
20	Dandelion, rutin, quercetin	*Taraxacum mongolicum* Hand.-Mazz.	H_2_O_2_ and UV induced hemolysis test	The activity of dandelion total flavonoids in H_2_O_2_ induced hemolysis test was stronger than that of rutin, quercetin, and positive control vitamin E. The activity of dandelion total flavonoids in UV induced hemolysis test slightly weaker than that of positive control vitamin E. Dandelion flavonoids had strong activity in scavenging ROS.	[92]
21	Total flavonoids (TOFs)	*Taraxacum officinale*	DPPH, ABTS, CB, FTC	In the DPPH assay, the IC_50_ values of TOFs, BHT, and vitamin C were 180.11 ± 7.85, 69.13 ± 4.32, and 77.98 ± 3.68 μg/mL, respectively. In the ABTS assay, the IC_50_ values of TOFs, BHT, and vitamin C were 10.18 ± 1.07, 2.02 ± 0.18, and 1.92 ± 0.04 μg/mL, respectively. In the CB assay, the lipid peroxidation inhibitions of TOFs, BHT, and vitamin C were 55.78%, 96.37%, and 4.30%, respectively. In the FTC assay, TOFs presented a strong antioxidant activity, but it was less efficient than BHT.	[101]
22	70% Ethanol extracts	*Taraxacum officinale*	O_2_^•−^, TBA	The hydroalcoholic extract of *T. officinale* exhibited the most effective radical scavenging ability at 830.78 µM TE/g, dw. And it was effective in protecting liposomes from lipid peroxidation with the IC_50_ value of 98.49 ± 6.67 µg/mL.	[91]
23	Methanolic extract	*Taraxacum offcinale* (L.)	DPPH, TRP, TAC	The methanolic extract revealed the highest DPPH activity (IC_50_, 32.80 ± 9.66 µg/mL), reducing potential (0.53 ± 0.02 mg/g), and TAC (19.42 ± 0.97 mg/g) as compared to the acetone and *n*-hexane extracts.	[102]
24	Water extract formula (WEF) derived from three TCM herbs	*Taraxacum officinale*	DPPH, ABTS, FRAP	In the DPPH assay, the IC_50_ values of water extract from *T. officinale* and WEF were 0.41 ± 0.02 and 0.39 ± 0.02 mg/mL, respectively. In the ABTS assay, Trolox equivalent antioxidant capacities of water extract from *T. officinale* and WEF were 276.7 ± 45.8 and 454.2 ± 15.7 mM/mg, respectively. In the FRAP assay, ascorbic acid equivalent antioxidant capacities of water extract from *T. officinale* and WEF were 536.1 ± 49.0 and 485.1 ± 50.9 mM/mg, respectively.	[103]
25	70% Ethanol extracts	*Taraxacum officinale*	DPPH, ABTS, FRAP	The extract of leaves showed higher antioxidant potential compared to the extract of roots for all parameters measured. The extract of leaves possessed a significantly (*p* < 0.01) higher DPPH (EC_50_ 0.37 compared to 1.34 mg/mL), ABTS (407.5 ± 0.14 compared to 171.5 ± 1.01 µM TE/mg of extract) and FRAP (156 ± 5.28 compared to 40 ± 0.3 µg VC/mg extract) capacities than the extract of roots.	[104]
26	Methanol extracts	*Taraxacum officinale*	TBA, O_2_^•−^	Leaves and petals 50% methanol fractions could be a new source of natural compounds showing cooperative activities: antioxidant, anti-platelet and anticoagulant, beneficial in the prevention and treatment of cardiovascular diseases, which are often associated with changes of hemostasis and oxidative stress.	[106]

Note: DPPH: 1,1-diphenyl-2-picrylhydrazyl; ABTS: 2,2′-azinobis-(3-ethylbenzthiazoline)-6-sulfonic acid; FRAP: total ferric-reducing antioxidant power;·OH: hydroxyl radical; O_2_^•−^: superoxide anion radical; CB: *β*-carotene bleaching assay; FTC: ferrothiocyanate assay; TBA: thiobarbituric acid assay; EC_50_: concentration for 50% of maximal effect; IC_50_: concentration for 50% of maximal inhibition; BHT: butylated hydroxytoluene; T-AOC: total antioxidant capacity; SOD: superoxide dismutase; GSH-Px: glutathione peroxidase; CAT: catalase; ROS: reactive oxygen species; UV: ultraviolet; dw: dry weight; H_2_O_2_: hydrogen peroxide; TE: Trolox; VC: ascorbic acid; *Taraxaci Herba*: the common name of dandelion in the *Pharmacopoeia of the People’s Republic of China*, and the accurate Latin name was not mentioned in the literature; *: extracts or compounds were acquired from elsewhere rather than experiments in the literature.

### 4.2. In Vivo Antioxidant Activities and Mechanisms

In vitro antioxidant activities’ studies of dandelion flavonoids have the advantages of low cost and easy operation, and the digestion, absorption, and metabolism of dandelion flavonoids by the body during normal metabolism, which introduce certain partialities and uncertainties. Therefore, it is necessary to further combine in vivo antioxidant activities’ studies to accurately evaluate their antioxidant activities. As shown in Table 8, in vivo antioxidant activities’ studies of dandelion flavonoids typically use representative animal models. The main animal models reported in the literature for in vivo antioxidant activities’ studies of dandelion flavonoids mainly include mouse models of chronic obstructive pulmonary disease (COPD) induced by tobacco smoke [96], mouse models of aging induced by D-galactose [108], rat models of liver injury induced by carbon tetrachloride (CCl_4_) [109], rat models of liver injury induced by sodium dichromate [87], lipopolysaccharide (LPS)-induced inflammation models in *Channa argus* [110], etc.

#### 4.2.1. Regulating mRNA Levels of Antioxidant Genes to Improve Antioxidant Capacity

In previous studies, dandelion flavonoids were proved to significantly increase the activities of SOD, GSH-Px, and T-AOC in mouse serum and liver tissue, reduce the content of malondialdehyde (MDA), and upregulate the mRNA expression of *SOD-1*, *SOD-2*, *GPX-1*, *GPX-4* mRNA. Dandelion flavonoids could improve the antioxidant capacities of mice and have anti-aging effects [111]. A study also reported that dandelion total flavonoid extracts could upregulate the mRNA levels of antioxidant genes (*Nrf2* and *SOD1*), regulate the expression levels of proteins related to the *Nrf2* signaling pathway, and play a protective role in COPD induced by cigarette smoke by regulating the *Nrf2* antioxidant signaling pathway [96]. According to literature reports, adding 50 mg/kg or 100 mg/kg *T. mongolicum* flavonoids to the diet could significantly increase T-AOC, CAT, and ascorbic acid (ASA) levels in the intestine of LPS-induced *C. argus*, as well as T-AOC, SOD, CAT, GSH-Px, glutathione reductase (GR), and ASA levels in the liver and pancreas, significantly reducing MDA and protein carbonyl (PC) levels. Adding 100 mg/kg *T. mongolicum* flavonoids to the diet could significantly upregulate the expression of antioxidant-related genes (*nrf2*, *gpx*, *gst*, *cat*) and heat shock proteins (*hsp70*, *hsp90*). *T. mongolicum* flavonoids could protect *C. argus* from LPS-induced inflammatory damage, improve antioxidant status, and suggest that *T. mongolicum* flavonoids can be added as an antioxidant to aquatic animal feed [110].

**Table 8:** In vivo antioxidant activities of flavonoids extracted from dandelion.

No.	Extracts/Compounds	Sources	Experimental Subjects	Results	References
1	95% Dandelion flavonoids	Nanjing Daosifu Biotechnology Co., Ltd. (Nanjing, China) *	5-Week-old SPF ICR male mice	Dandelion flavonoids could make the activities of SOD, GSH-Px, and T-AOC in mouse serum and liver tissue significantly increased, the content of MDA reduced significantly and the expression of *SOD-1*, *SOD-2*, *GPX-1*, *GPX-4* mRNA increased significantly. Dandelion flavonoids could improve the antioxidant capacity of mice and had anti-aging effects.	[111]
2	Dandelion flavonoids extracts	Research office of natural drug research center, school of pharmacy, Jilin university *	COPD induced by cigarette smoke (female SPF-grade BALB/c mice)	Dandelion total flavonoids could upregulate the mRNA levels of antioxidant genes (*Nrf2* and *SOD1*), could regulate the expression levels of proteins related to the *Nrf2* signaling pathway, might play a protective role in COPD induced by cigarette smoke by regulating *Nrf2* antioxidant signaling pathway.	[96]
3	Anti-aging dandelion health product composition (include dandelion flavonoids)	Jiangsu Yichao Biotechnology Co., Ltd. (Huaian, China) *	Aging mice caused by D-galactose	The active ingredient composition of the health product could significantly reduce the content of MDA in aging mice caused by D-galactose, and could significantly improve the activities of CAT, SOD, T-AOC, GSH-Px enzymes in serum and brain tissue of aging mice. The health product composition could be used as anti-aging drugs or anti-aging health products.	[108]
4	Dandelion total flavonoids	Xian Aoruite Biotechnology Co., Ltd. (Xi’an, China) *	CCl_4_ induced liver injury in Wistar rats	Dandelion total flavonoids could increase the levels of SOD and GSH-Px, and reduce the levels of MDA in rat liver tissue, and improve pathological damage in rat liver tissue.	[109]
5	Dandelion leaf extracts (total flavonoids)	*Taraxacum officinale* Weber	Sodium dichromate-induced liver injury in rats	The levels of SOD, CAT, GSH-Px in the liver of sodium dichromate-induced liver injury rats increased after adding dandelion leaf extract, while the levels of MDA significantly decreased.	[87]
6	Dandelion flavonoids	Shanxi Jinkangtai Biotechnology Co., Ltd. (Xixianxinqu, China) *	LPS-induced *Channa argus*	Adding 50 mg/kg or 100 mg/kg dandelion flavonoids to the diet could significantly increase T-AOC, CAT, and ASA levels in the intestine of LPS induced *C. argus*, as well as T-AOC, SOD, CAT, GSH-Px, GR, and ASA levels in the liver and pancreas, and significantly reduce MDA and PC levels. Adding 100 mg/kg dandelion flavonoids to the diet could significantly upregulate the expression of antioxidant related genes (*nrf2*, *gpx*, *gst*, *cat*) and heat shock proteins (*hsp70*, *hsp90*). Dandelion flavonoids could protect *C. argus* from lipopolysaccharide induced inflammatory damage, improve antioxidant status, and suggest that dandelion flavonoids can be added as an antioxidant to aquatic animal feed.	[110]
7	Dandelion extract (polysaccharides 5.09%, flavonoids 2.15%)	A certain biotechnology company (batch number: BWPGY20210907) *	Male Kebao-500 broilers	The activity of GSH-Px in 21-day-old broilers treated with dandelion extract significantly increased. Adding 0.1% dandelion extract alone to the diet could enhance the immune function, improve antioxidant capacity, and thus improve growth performance of broilers. Moreover, the simultaneous addition of danshen extract and dandelion extract was more effective than adding them alone.	[112]
8	Water extract formula (WEF) derived from three TCM herbs	*Taraxacum officinale*	CCl_4_ induced hepatic damage in rats	The animal experiments revealed that the WEF administration could lower MDA and GSH levels, and reform or resume SOD content as well as improve GSH-Px, GR and CAT activities in CCl_4_ induced rats.	[103]
9	70% Ethanol extracts	*Taraxacum officinale*	*Nω*-nitro-_L_-arginine methyl ester induced hypertensive rats	The extract of leaves and the extract of roots significantly reduced MDA levels in targets organs.	[104]

Note: SPF: specific pathogen free; ICR: institute of cancer research; LPS: lipopolysaccharide; COPD: chronic obstructive pulmonary disease; T-AOC: total antioxidant capacity; SOD: superoxide dismutase; GSH: glutathione; GSH-Px: glutathione peroxidase; GR: glutathione reductase; CAT: catalase; MDA: malondialdehyde; ASA: ascorbic acid; PC: protein carbonyl; *: extracts or compounds were acquired from elsewhere rather than experiments in the literature.

#### 4.2.2. Regulating Antioxidant Enzyme Activities to Improve Antioxidant Capacity

In the literature, the active ingredient composition of the health product, which included effective components extracted from dandelion, chicory root, and perilla leaf, could significantly reduce the content of MDA in aging mice caused by D-galactose and could significantly improve the activities of CAT, SOD, T-AOC, GSH-Px enzymes in the serum and brain tissue of aging mice. The health product composition was proved to be used as anti-aging drugs or anti-aging health products [108]. Dandelion total flavonoids were proved to increase the levels of SOD and GSH-Px, reduce the levels of MDA in rat liver tissue, and improve pathological damage in rat liver tissue [109]. In a certain study, it was indicated that the levels of SOD, CAT, and GSH-Px in the liver of sodium dichromate-induced liver injury rats increased after adding dandelion leaf extract, while the levels of MDA significantly decreased. Dandelion leaf extract showed potential in the protection against sodium dichromate-induced hepatotoxicity, which was consistent with the results of the DPPH assay [87]. As shown in the literature, the activity of GSH-Px in 21-day-old broilers treated with dandelion extract significantly increased. Adding 0.1% dandelion extract alone to the diet could enhance the immune function, improve antioxidant capacity, and, thus, improve the growth performance of broilers. Moreover, the simultaneous addition of Danshen extract and Dandelion extract was more effective than adding them alone [112]. The animal experiments revealed that the water extract formula derived from *M. stigma*, *N. nucifera* Gaertn and *T. officinale* administration could lower MDA and GSH levels and reform or resume SOD content as well as improve GSH-Px, GR and CAT activities in CCl_4_-induced rats [103]. The study found that the extract of leaves and the extract of roots from *T. officinale* significantly reduced MDA levels in targets organs [104].

## 5. Potential Application

As a well-known traditional Chinese medicine, dandelion encompasses a diverse range of functional flavonoids. Numerous domestic and international reports have unveiled the remarkable antioxidant effects of flavonoid-containing dandelion extracts and pure flavonoids derived from dandelion. As shown in Table 9, these findings supplied a solid foundation for the potential application of dandelion in the fields of medicine [28,64,66,70,78,80,81,87,96,102,103,104,106,108,109], functional foods and drinks [15,68,90], feeds [3,12,14,73,88,110,112], and cosmetics [13].

**Table 9:** Potential application related to dandelion flavonoids and their antioxidant activities.

No.	Flavonoids-Containing Extracts	Major Flavonoids Identified from Extracts	Potential Application	Effects	References
1	50% ethanol extract of dandelion	**4**, **6**, **12**, **62**, **87**, **111**	medicine	anticancer	[28]
2	dandelion extract	**103**, **150**	medicine	anticancer	[78]
3	not mentioned	**4**, **21**, **43**, **44**	medicine	*α*-glucosidase inhibitory	[64]
4	60% hydroethanolic extract of dandelion	**8**, **31**, **73**, **91**, **95**, **99**, **100**	medicine	antioxidant, anti-obesity	[66]
5	dandelion extract	**3**, **5**, **11**, **29**, **30**, **44**, **63**, **78**, **84**, **92**, **108**, **142**, **144**, **146**, **147**, **149**	medicine	antioxidant, hepatoprotective	[70]
6	hydro-methanolic extract of dandelion	**49**, **89**, **108**, **111**	medicine	antimicrobial	[80]
7	hydromethanolic extract of dandelion	**49**, **89**, **108**, **111**	medicine	antidepressant	[81]
8	dandelion leaf extract	not mentioned	medicine	hepatoprotective	[87]
9	total flavonoids from dandelion	**4**, **6**, **12**, **21**, **28**, **62**, **83**, **84**	medicine	antioxidant	[96]
10	dandelion methanolic extract	not mentioned	medicine	antioxidant, cytotoxic, and phytotoxic	[102]
11	formula derived from *Maydis stigma*, *Nelumbo nucifera* and dandelion	not mentioned	medicine	antioxidant and hepatoprotective	[103]
12	dandelion leaf extract	not mentioned	medicine	antioxidant	[104]
13	dandelion leaves and petals 50% methanol fractions	not mentioned	medicine	antioxidant and anticoagulant	[106]
14	anti-aging dandelion health product	not mentioned	drug or health product	anti-aging	[108]
15	dandelion flavonoids	not mentioned	medicine	hepatoprotective	[109]
16	dandelion fermented stock solution	**4**, **62**, **84**	beverage	antioxidant	[15]
17	crude extract from dandelion and fermented dandelion	**2**, **35**, **38**, **60**, **72**, **84**, **109**, **115**, **116**, **117**, **118**, **122**, **124**, **126**, **135**, **136**, **138**, **140**, **141**, **155**	food additive	antioxidant	[68]
18	95% ethanol and subsequent water extracts from dandelion roots	not mentioned	food additive	antioxidant	[90]
19	dandelion water extract added in basic feed	not mentioned	feed	increase milk production, milk protein content, and milk fat content in cows, reduce somatic cell count in milk, increase fecal microbial diversity and relative abundance, and positively regulate the body’s antioxidant capacity and immune function	[3]
20	dandelion grass powder added in basic feed	not mentioned	feed	reduce the incidence rate and mortality of broilers	[12]
21	dandelion flavonoids added in basic feed	not mentioned	feed	antioxidant, enhance the immune function and improve growth performance of broilers	[112]
22	dandelion flavonoids add in *Caragana korshinskii* silage	not mentioned	feed	antioxidant, improve nutritional and quality and fermentation quality, optimize the microbial community structure, promote the growth of beneficial bacteria, and inhibit the growth of bad bacteria	[14]
23	2% total flavonoids from dandelion	not mentioned	feed	promote the fermentation quality and antioxidant activity of *Caragana korshinskii* Kom. silage	[88]
24	flavonoids from fermented dandelion	**16**, **37**, **44**, **47**, **48**, **56**, **57**, **58**, **68**, **71**, **92**, **93**, **94**, **98**, **119**, **139**	feed additive	antioxidant	[73]
25	dandelion flavonoids	not mentioned	feed additive	antioxidant, anti-inflammatory	[110]
26	complexes of dandelion extract and *Salvia miltiorrhiza* volatile oil	not mentioned	cosmetic	antioxidant, antibacterial, no obvious cytotoxicity, better skin permeability	[13]

### 5.1. Potential Application in Medicine

The literature shows that flavonoid-containing dandelion extracts or pure flavonoids derived from dandelion possess significant antioxidant activities and could be potentially used as antitumor [28,78], hypoglycemic [64], hypolipidemic [66], hepatoprotective [70,87,103,109], antihypertensive [104], anticoagulant [106], anti-aging [108], antibacterial [80], and antidepressant drugs or health products [81].

One study showed that a 50% ethanol extract of *T. mongolicum,* which was rich in flavonoids, such as luteolin (**4**), luteolin-7-*O*-*β*-D-glucoside (**6**), luteolin-7-*O*-*β*-D-rutinoside (**12**), quercetin (**62**), isorhamnetin-3-*O*-*β*-D-glucoside (**87**), and hesperidin (**111**), possessed the possible therapeutic potential for triple-negative breast cancer by inducing G2/M phase arrest and activating apoptosis in MDA-MB-231 cells through ER stress [28]. It was not unique; other research results concluded that dandelion extract, which was identified as containing 2-(3,4-dihydroxy-5-methoxyphenyl)-3,5,7-trihydroxy-6-methoxy-4*H*-chromen-4-one (**103**) and mangostenone B (**150**), might exert anticancer activity by inducing A549 cell death with complex mechanisms [78]. There was also a study that found luteolin (**4**), chrysoeriol (**21**), 5,7,3′-trihydroxy-4′,5′-dimethoxy flavone (**43**), and tricin (**44**) isolated from *T. officinale* displayed outstanding *α*-glucosidase inhibitory activities as potential novel potent α-glucosidase inhibitors, with IC_50_ values of 39.8 ± 4.2 μM, 155.9 ± 3.2 μM, 154.1 ± 2.5 μM, and 161.6 ± 2.2 μM, respectively [64]. There was also a study that presented a 60% hydroethanolic extract of *T. officinale* as a potential source of antioxidant and anti-obesity ingredients. Flavonoids identified from a 60% hydroethanolic extract of *T. officinale,* such as luteolin-7-*O*-*β*-D-(6″-acetyl)-glucopyranoside (**8**), isovitexin-3″-*O*-glucopyranoside (**31**), myricetin (**73**), kaempferol-3-*O*-*β*-D-glucopyranoside (**91**), kaempferol-3-*O*-rhamnoside (**95**), kaemperfol-3,7-diglucoside (**99**), and kaempferol-3-*O*-*β*-D-glucoside-7-*O*-*α*-L-arabinofuranoside (**100**), showed considerable binding affinity with pancreatic lipase [66]. Additionally, *Taraxaci extractum* extract, which was identified as containing 6,2′-dihydroxyflavone (**3**), luteolin-7-*O*-glucoside (**5**), luteolin-7-*O*-rutinose (**11**), apigenin-7-*O*-glucuronide (**29**), vitexin (**30**), tricin (**44**), quercetin-3-*O*-glucoside (**63**), quercetin-3-*O*-glucuronide (**78**), rutin (**84**), kaempferol-3-*O*-rutinoside (**92**), naringenin (**108**), genistein (**142**), tectorigenin (**144**), pseudobaptigenin (**146**), formononetin (**147**), and genistin (**149**), exerted antioxidant and hepatoprotective properties to prevent oxidative damage and inflammation in human liver cells [70]. Similarly, there were also findings powerfully supporting the fact that the *T. officinale* leaf extract was effective in the protection against liver injury in rats [87]. In addition, the formula derived from *Maydis stigma*, *Nelumbo nucifera*, and *T. officinale* showed antioxidant and hepatoprotective effects on liver damage [103]. There was a study concluding that dandelion flavonoids showed a certain protective effect in the early stage of liver injury [109]. Containing luteolin (**4**), luteolin-7-*O*-*β*-D-glucoside (**6**), luteolin-7-*O*-*β*-D-rutinoside (**12**), luteolin-3′-methyl ether (**21**), apigenin-7-*O*-glucoside (**28**), quercetin (**62**), quercetin-3′,4′,7-trimethyl ether (**83**), and rutin (**84**), total flavonoids from dandelion were found to regulate the Nrf2 antioxidant signaling pathway to play a protective role in chronic obstructive pulmonary disease [96]. What’s more, the *T. officinale* methanolic extract exhibited efficiency on antioxidant, cytotoxic, and phytotoxic potential [102]. In another study, the *T. officinale* leaf extract possessed antioxidant protection against hypertensive rats [104]. Notably, *T. officinale* leaves and petals’ 50% methanol fraction was proven to possess antioxidant and anticoagulant activity, which had potential in the treatment of cardiovascular diseases [106]. Furthermore, an anti-aging dandelion health product composition prepared with dandelion, chicory root, and perilla leaf was demonstrated to show significant antioxidant activity, indicating that dandelion could be used in anti-aging drugs or anti-aging health products [108]. Moreover, the hydro-methanolic extract of *T. mongolicum,* which was rich in flavonoids, such as isoetin (**49**), kaempferol (**89**), naringenin (**108**), and hesperidin (**111**), and phenolic compounds exhibited significant antimicrobial activity against respiratory tract bacterial strains [80]. In addition, a hydromethanolic extract of *T. officinale* exerted antidepressant effects by inhibiting corticosterone levels and modulating the expression of mitogen-activated protein kinase phosphatase-1 and brain-derived neurotrophic factor. In addition, the bioactive flavonoids, which were potentially responsible for the antidepressant effects of *T. officinale* hydromethanolic extract, were identified as isoetin (**49**), kaempferol (**89**), naringenin (**108**), and hesperidin (**111**) [81].

### 5.2. Potential Application in Functional Foods

Studies have shown that flavonoid-containing dandelion extracts or pure flavonoids derived from dandelion possessed significant antioxidant activities and could be potentially used to produce a dandelion fermented beverage [15] and food additive [68,90].

Based on the antioxidant activities of luteolin (**4**), quercetin (**62**), and rutin (**84**) derived from dandelion, a kind of fermented dandelion beverage, which was beneficial to human health, was finally obtained [15]. In particular, flavonoids, such as 7,4′-dihydroxyflavone (**2**), baicalein (**35**), pedalitin (**38**), 3-hydroxyflavone (**60**), myricitrin (**72**), rutin (**84**), naringenin-7-*O*-glucoside (**109**), liquiritigenin (**115**), liquiritin (**116**), farrerol (**117**), garbanzol (**118**), dihydromyricetin (**122**), cyanidin (**124**), delphinidin-3-*O*-glucoside (**126**), butein (**135**), xanthohumol (**136**), isoliquiritigenin (**138**), daidzein (**140**), 2-hydroxyxydaidzein (**141**), and amentoflavone (**155**), were different between dandelion and fermented dandelion. Furthermore, solid-state fermentation could effectively enhance the content of dandelion flavonoids and their antioxidant capacities. In addition, a crude extract from fermented dandelion displayed significant antioxidant activity, which could be applied as an antioxidant and functional food additive [68]. Moreover, 95% ethanol and subsequent water extracts from *T. officinale* roots showed significant antioxidant activities, which demonstrated that the *T. officinale* root is valuable for use as natural antioxidants in foods [90].

### 5.3. Potential Application in Feed

Previous experiments have shown that flavonoid-containing dandelion extracts or pure flavonoids derived from dandelion possessed significant antioxidant activities and could be potentially added to feed [3,12,14,73,88,110,112]. It was found that adding dandelion water extract in basic feed could increase milk production, milk protein rate, and milk fat rate in cows; reduce somatic cell count in milk; increase fecal microbial diversity and relative abundance; and positively regulate the body’s antioxidant capacity and immune function [3]. A study also indicated that the addition of dandelion powder to basic feed could reduce the incidence rate and mortality of broilers [12]. Additionally, the addition of dandelion flavonoids to basic feed could improve the antioxidant properties and immunity of broilers [112]. A study indicated that the addition of dandelion powder to basic feed could reduce the incidence rate and mortality of broilers [112]. Moreover, it was also found that different amounts of dandelion flavonoids could improve the antioxidant activity, nutritional quality, fermentation quality, microbial community structure, and the growth of beneficial bacteria of *C. korshinskii* silage [14]. Furthermore, *C. korshinskii* silage ensiled with 2% total flavonoids from *T. mongolicum* Hand.-Mazz add was proven to possess good fermentation quality and antioxidant activity [88]. In addition, a study found that major flavonoids from fermented dandelion, including lonicerin (**16**), hispidulin (**37**), tricin (**44**), jaceosidin (**47**), tangeretin (**48**), homoorientin (**56**), isoscutellarein (**57**), tetrahydroxyflavonoe-*C*-rhamnosyl-glucoside (**58**), avicularin (**68**), quercetin-3-*O*-*α*-L-rhamnopyranoside (**71**), kaempferol-3-*O*-rutinoside (**92**), kaempferol-3-*O*-neohesperiidoside (**93**), kaempferol-3-*O*-robinobioside (**94**), kaempferol-3-*O*-glucoside-7-*O*-rhamnoside (**98**), toxifolin (**119**), and phloretin (**139**), displayed superior potential as a natural antioxidant in animal husbandry for its good antioxidant activity [73]. One report elucidated that the dietary supplementation of *T. mongolicum* flavonoids was proven to possess significant anti-inflammatory and antioxidant activities [110].

### 5.4. Potential Application in Cosmetics

In addition, the complexes of *Salvia miltiorrhiza* volatile oil and dandelion extract showed synergistic antioxidant and antibacterial effects, illustrating great potential in cosmetics [13].

## 6. Conclusions and Prospects

In conclusion, dandelion has broad application prospects in the fields of pharmaceuticals, foods, health products, daily chemicals, and feed additives, but current correlational research on dandelion antioxidant activities, bioactive constituents, and underlying mechanisms still needs improvement. Firstly, research on the flavonoids of dandelion is not systematic and comprehensive enough. Currently, only over 150 types of flavonoids are identified from dandelion, requiring further exploration. Secondly, most current studies on the antioxidant activities of dandelion flavonoids are limited to crude extracts or dandelion total flavonoids, and there is a lack of research on the antioxidant activities and mechanisms of action of flavonoids. Therefore, research on the antioxidant activities of flavonoids extracted and isolated from other plants can provide a reference for the study of the antioxidant activities and mechanisms of action of dandelion flavonoids. Thirdly, in vitro antioxidant activities’ studies of dandelion flavonoids exhibit certain partialities and inaccuracies. Even combining various in vitro antioxidant activities cannot accurately reflect the actual utilization value of dandelion flavonoids. The digestion, absorption, distribution, metabolism, and bioavailability of dandelion flavonoids affect their antioxidant activities and require further in-depth research. Moreover, in cellular or in vivo antioxidant activity studies of dandelion flavonoids, there are reports on the regulation of mRNA levels of antioxidant genes and the activity of antioxidant enzymes by dandelion flavonoids. However, specific regulatory mechanisms, such as whether dandelion flavonoids regulate gene expression through transcription factors or directly interact with nucleic acids and the cross-effects of dandelion flavonoids among various cellular pathways in oxidative stress, have not been fully elucidated. Finally, there are relatively few studies on the structure–activity relationships of the antioxidant activities of dandelion flavonoids, and most existing studies focus on the ability to scavenge free radicals. Reports on other mechanisms by which dandelion flavonoids exert antioxidant effects, such as regulating mRNA levels of antioxidant genes, regulating antioxidant enzyme activity, and regulating oxidative stress-related signaling pathways, are even fewer. These may become important directions for future research on the antioxidant mechanisms of dandelion flavonoids. Research on dandelion flavonoids and their antioxidant activities will become more detailed, and the research and development of dandelion flavonoids will reach a higher level due to the rapid development and interdisciplinary integration of disciplines, such as biochemistry, molecular biology, cell biology, bioinformatics, and phytochemistry.
